# Canonical PI3Kγ signaling in myeloid cells restricts *Trypanosoma cruzi* infection and dampens chagasic myocarditis

**DOI:** 10.1038/s41467-018-03986-3

**Published:** 2018-04-17

**Authors:** Maria C. Silva, Marcela Davoli-Ferreira, Tiago S. Medina, Renata Sesti-Costa, Grace K. Silva, Carla D. Lopes, Lucas E. Cardozo, Fábio N. Gava, Konstantina Lyroni, Fabrício C. Dias, Amanda F. Frade, Monique Baron, Helder I. Nakaya, Florêncio Figueiredo, José C. Alves-Filho, Fernando Q. Cunha, Christos Tsatsanis, Christophe Chevillard, Edecio Cunha-Neto, Emilio Hirsch, João S. Silva, Thiago M. Cunha

**Affiliations:** 1 Department of Pharmacology, Ribeirão Preto Medical School, University of Sao Paulo, Bandeirantes Avenue, 3900, Ribeirão Preto, SP 14049-900 Brazil; 2 Department of Biochemistry and Immunology, Fiocruz- Bi-Institutional Translational Medicine Plataform, Ribeirão Preto Medical School, University of Sao Paulo, Bandeirantes Avenue, 3900, Ribeirão Preto, SP 14049-900 Brazil; 3 Department of Clinical and Toxicological Analyses, School of Pharmaceutical Sciences, University of São Paulo, São Paulo, SP 05508-900 Brazil; 4 Department of Physiology, Ribeirão Preto Medical School, University of Sao Paulo, Bandeirantes Avenue, 3900, Ribeirão Preto, SP 14049-900 Brazil; 5 Laboratory of Clinical Chemistry, School of Medicine, University of Crete, 71003 Heraklion, Greece; 6 Laboratory of Immunology, Heart Institute (InCor), School of Medicine, University of São Paulo, São Paulo, SP 05403-900 Brazil; 7 Laboratory of Pathology, School of Medicine, University of Brasilia, Campus Universitário Darcy Ribeiro, Brasilia, DF 70910-900 Brazil; 8 TAGC/INSERM U1090, Aix Marseille Université AMU, Parc Scientifique de Luminy case 928, 163 Avenue de Luminy, 13288 Marseille, Cedex 09 France; 9 Division of Clinical Immunology and Allergy, School of Medicine, University of São Paulo, São Paulo, SP 05403-900 Brazil; 10 Institute of Investigation in Immunology-iii/INCT, São Paulo, SP 05403-900 Brazil; 11 Molecular Biotechnology Center, Department of Molecular Biotechnology and Health Sciences, University of Torino, Turin, 10126 Italy; 12 Present Address: Post Graduation Program in Biotechnology, Universidade Católica Dom Bosco, Campo Grande, MS Brazil; 13 Present Address: Department of Bioengineering, Brazil University, Rua Carolina da Fonseca, 234 (Campus II), Itaquera, São Paulo, SP 08230-030 Brazil

## Abstract

Chagas disease is caused by infection with the protozoan *Trypanosoma cruzi* (*T*. *cruzi*) and is an important cause of severe inflammatory heart disease. However, the mechanisms driving Chagas disease cardiomyopathy have not been completely elucidated. Here, we show that the canonical PI3Kγ pathway is upregulated in both human chagasic hearts and hearts of acutely infected mice. PI3Kγ-deficient mice and mutant mice carrying catalytically inactive PI3Kγ are more susceptible to *T*. *cruzi* infection. The canonical PI3Kγ signaling in myeloid cells is essential to restrict *T*. *cruzi* heart parasitism and ultimately to avoid myocarditis, heart damage, and death of mice. Furthermore, high *PIK3CG* expression correlates with low parasitism in human Chagas’ hearts. In conclusion, these results indicate an essential role of the canonical PI3Kγ signaling pathway in the control of *T*. *cruzi* infection, providing further insight into the molecular mechanisms involved in the pathophysiology of chagasic heart disease.

## Abstract (web summary)

*Trypanosoma cruzi* infection causes Chagas disease, but mechanisms underlying pathogenesis are unclear. Here, Silva et al. show that canonical PI3Kγ signaling in myeloid cells restricts *T*. *cruzi* infection in mice and that high *PIK3CG* expression correlates with low parasite levels in human Chagas’ hearts.

## Introduction

Chagas disease is caused by infection with the intracellular protozoan parasite *Trypanosoma cruzi* (*T*. *cruzi*). Currently, approximately 6–7 million people worldwide, mostly in Latin America, are infected^1,2^. At present, due to migration to North America, Europe, and Japan, it is a global health concern^3–6^. The disease is characterized by an acute phase, usually asymptomatic and with evident parasitemia detectable in the bloodstream^7^. In most individuals, manifestations of the acute phase disappear a few weeks after infection^8^, followed by the chronic phase of infection. Although the chronic phase of Chagas disease is most likely featured by the asymptomatic form, approximately 30% of patients develop the cardiac form^9^. Life-threatening chronic chagasic cardiomyopathy (CCC) is the most severe manifestation of the disease, which is characterized by an intense Th1 T-cell and macrophage-rich myocarditis, destruction of myofibers, and alteration of cardiac function^10,11^. It can lead to refractory heart failure and sudden death in a significant proportion of patients. The molecular mechanisms involved in the pathophysiology of chagasic heart disease are unclear.

Recognition of the parasite by resident and recruited leukocytes activates several signaling pathways, including phosphatidylinositol 3-kinases (PI3Ks) signaling, which is involved in different cellular processes through the phosphorylation of lipids and proteins^12,13^. Among the PI3K enzyme family, PI3Kγ signaling is activated by the βγ subunit of G-protein-coupled receptors and plays an important role in leukocyte functions, inflammation, and heart dysfunction^14^.

Here, we show that the canonical PI3Kγ is involved in the pathophysiology of chagasic heart disease. The canonical PI3Kγ pathway is upregulated in both human chagasic hearts and hearts of acutely infected mice. Deficient mice in the canonical PI3Kγ signaling are more susceptible to *T*. *cruzi* infection. The canonical PI3Kγ signaling in myeloid cells restricts heart parasitism and avoids heart damage and death of mice. Furthermore, high *PIK3CG* expression correlates with low parasitism in human chagasic hearts. These data identify a previously unrecognized role of the canonical PI3Kγ signaling pathway in the control of *T*. *cruzi* infection, providing further insight into the molecular mechanisms involved in chagasic heart disease.

## Results

### Canonical PI3Kγ signaling protects from *T*. *cruzi* infection

**Fig. 1:**
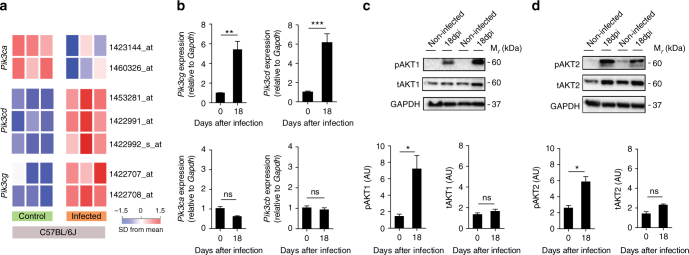
The PI3Kγ signaling is enhanced in the heart tissue of mice after experimental infection with *T*. *cruzi*. **a** Transcriptome analysis for the expression of *Pik3ca*, *Pik3cd*, and *Pik3cg* genes in non-infected C57BL/6J mice or 18 days post infection with *T*. *cruzi*. **b** RT-PCR analysis of the mRNA expression of *Pik3ca*, *Pik3cb*, *Pik3cd*, and *Pik3cg* genes in the heart tissue of C57BL/6 non-infected mice (*n* = 7) or 18 days post infection with *T*. *cruzi* Y strain (*n* = 11). *Gapdh* was used as a housekeeping gene. **c** Representative western blots and analysis of phosphorylated (p) and total (t) AKT1 expression in the heart tissue of non-infected C57BL/6 mice or 18 days post infection with *T*. *cruzi* (*n* = 6). GAPDH was used as a loading control. **d** Representative western blots and analysis of phosphorylated (p) and total (t) AKT2 expression in the heart tissue of non-infected C57BL/6 mice or 18 days post infection with *T*. *cruzi* (*n* = 6). GAPDH was used as a loading control. AU refers to arbitrary units (**c**, **d**). *P < 0.05, **P < 0.01, ***P < 0.001, and ns = no statistical significance (unpaired Student’s *t-*test in **b**–**d**). Data are representative of two (**b**–**d**) independent experiments (mean ± s.e.m in **b**–**d**)

We initially examined the expression and activation of PI3Kγ signaling in the heart tissue of *T*. *cruzi*-infected mice. For this purpose, we performed analysis of the cardiac tissue transcriptome from *T*. *cruzi* Y strain-infected C57BL/6 mice previously obtained by our group^15^ (GSE41089). Data revealed that the expression of *Pik3cg* (*P* = 4.0E-3; unpaired *t*-test) and *Pik3cd* (*P* = 7.4E-6; unpaired *t*-test) genes, but not *Pik3ca*, are upregulated in the heart tissue of *T*. *cruzi*-infected mice 18 days post infection (dpi) (Fig. 1a). Corroborating our transcriptome analysis, heart tissue of infected C57BL/6 mice showed an increased mRNA expression of *Pik3cg* and *Pik3cd* genes, but not *Pik3ca*, compared to heart samples from uninfected mice (Fig. 1b). Following activation, PI3Kγ promotes downstream signaling through the protein kinase B (AKT) pathway^16^. The phosphorylated forms of AKT (pAKT) were thus studied as markers of the canonical PI3Kγ signaling activation. AKT is a family of kinases consisting of three isoforms of AKTs, AKT1, AKT2, and AKT3 encoded by independent genes. AKT1 and AKT2 are expressed in leukocytes and have been implicated in the immune response^17^. In this context, we sought to investigate whether PI3Kγ signaling was enhanced in the heart tissue of *T*. *cruzi-*infected mice through the measurement of pAKTs (pAKT1 and pAKT2). Although the infection does not change the expression levels of *Akt1* and *Akt2* mRNA (Supplementary Fig. 1a, b) and proteins (Fig. 1c, d), the phosphorylated forms of these proteins are upregulated in the heart tissue of infected mice compared to the uninfected group (Fig. 1c, d). These results indicate that after infection with *T*. *cruzi*, PI3Kγ signaling is upregulated in the heart of infected mice.

**Fig. 2:**
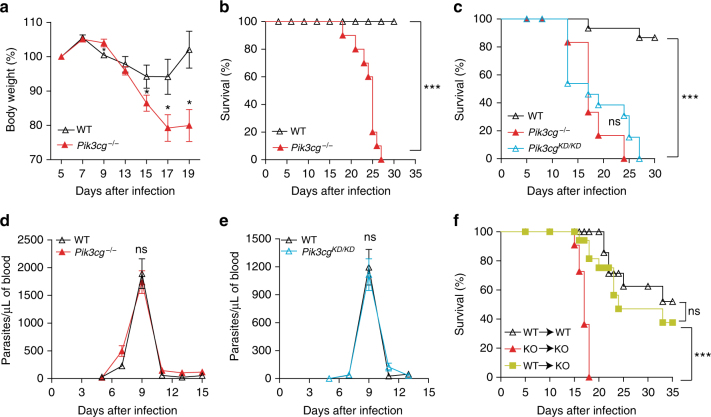
PI3Kγ signaling in hematopoietic cells is essential for mouse resistance to *T*. *cruzi* infection. **a**–**c** Body weight (**a**) and survival rate of C57BL/6 WT (*n* = 10) and *Pik3cg*^*−/−*^ (*n* = 10) (**b**) or *Pik3cg*^*KD/KD*^ (*n* = 10) (**c**) mice infected with 10^3^ trypomastigote forms of *T*. *cruzi* Y strain. **d**, **e** Blood parasitemia of WT and *Pik3cg*^*−/−*^ (**d**) or *Pik3cg*^*KD/KD*^ (**e**) mice infected with 10^3^ trypomastigote forms of *T*. *cruzi* Y strain. **f** Survival rate of chimera mice infected with 500 trypomastigote forms of *T*. *cruzi* Y strain. *P < 0.05, ***P < 0.001, and ns = no statistical significance (unpaired Student’s *t*-test in **a**, **d**, **e** and Mantel–Cox log-rank test in **b**, **c**, **f**). Data are representative of two (**f**) of three (**a**–**e**) independent experiments (mean ± s.e.m in **a**,** d**, **e**)

To evaluate whether PI3Kγ signaling pathways contribute for the pathogenesis of chagasic disease, we took advantage of the experimental mouse model of this disease. In this context, wild-type (WT) and *Pik3cg*^*−/−*^ mice were infected with *T*. *cruzi* Y strain. Unlike C57BL/6 WT mice, which had a slight reduction in their body weight during infection, *Pik3cg*^*−/−*^ mice presented a significant reduction in their body weight starting at 9th day after infection and progressively worsened (Fig. 2a). Moreover, differently of the WT-infected mice, which presented 100% of survival, all the infected *Pik3cg*^*−/−*^ mice succumbed until 30 dpi (Fig. 2b). Remarkably, heterozygous *Pik3cg*^*+/−*^ mice were protected from death caused by *T*. *cruzi* infection when compared with homozygous *Pik3cg*^*−/−*^ mice (Supplementary Fig. 2). Although biological activities of PI3Kγ have been ascribed mainly by its ability to convert PIP2 into PIP3, there is also evidence that PI3Kγ can act as a scaffold protein, independently of the kinase function^18^. In this context, we infected mutant mice carrying a catalytically inactive PI3Kγ (*Pik3cg*^*KD/KD*^)^18^. Similarly to *Pik3cg*^*−/−*^ mice, *Pik3cg*^*KD/KD*^ mice were also susceptible to *T*. *cruzi* infection (Fig. 2c). In agreement with genetic inhibition of the PI3Kγ catalytic activity, pharmacological inhibition of PI3Kγ activity in WT mice with a partial selective PI3Kγ isoform inhibitor, AS605240, increased the weight loss and mortality when compared to vehicle-treated mice (Supplementary Fig. 3a, b). Despite of having a high mortality rate, *Pik3cg*^*−/−*^ and *Pik3cg*^*KD/KD*^ mice showed similar numbers of circulating parasites in the blood across the whole time course compared to WT mice (Fig. 2d, e), suggesting that the inefficient control of the systemic parasitemia is not the cause of the increased *Pik3cg*^*−/−*^ and *Pik3cg*^*KD/KD*^ mice mortality. To evaluate in which cell type (cardiomyocytes or hematopoietic cells/leukocytes) PI3Kγ signaling is important for the pathophysiology of chagasic heart disease, bone marrow (BM) chimeric mice were generated. Interestingly, the transfer of BM from WT mice to irradiated *Pik3cg*^*−/−*^ mice protected recipient mice from *T*. *cruzi* infection-induced death (Fig. 2f). These data suggest that an integral PI3Kγ signaling pathway in hematopoietic cells/leukocytes is central for the control of *T*. *cruzi* infection.

### PI3Kγ protects from *T*. *cruzi* infection-induced cardiac damage

**Fig. 3:**
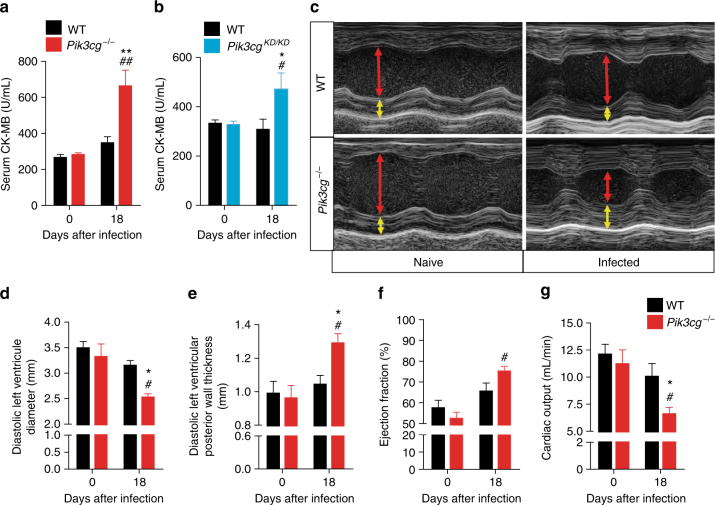
PI3Kγ is important for the heart function of *T*. *cruzi*-infected mice. **a**, **b** Serum CK-MB level of WT (*n* = 4), *Pik3cg*^*−/−*^ (*n* = 4) (**a**) and *Pik3cg*^*KD/KD*^ (*n* = 7) (**b**) non-infected mice or 18 days post infection with 10^3^ trypomastigote forms of *T*. *cruzi* Y strain. **c** Representative images of echocardiography in WT (*n* = 6) and *Pik3cg*^*−/−*^ (*n* = 6) non-infected mice or 18 days after infection with *T*. *cruzi*. Red arrows indicate diastolic ventricular diameter and yellow arrow indicates diastolic ventricular wall thickness. **d**–**g** Diastolic left ventricle diameter (**d**), diastolic left ventricular posterior wall thickness (**e**), ejection fraction (**f**), and cardiac output (**g**) of WT (*n* = 6) and *Pik3cg*^*−/−*^ (*n* = 6) non-infected mice or 18 days after infection with 10^3^ trypomastigote forms of *T*. *cruzi* Y strain. *P < 0.05 and **P < 0.01 relative to WT-infected mice and #*P* < 0.05 and ##*P* < 0.01 relative to non-infected group (one-way ANOVA with Tukey’s post hoc test in **a**, **b**, **d**–**g**). Data are representative of two independent experiments (mean ± s.e.m in **a**,** b**, **d**–**g**)

To elucidate the cause of the increased susceptibility to *T*. *cruzi* infection by *Pik3cg*^*−/−*^ mice, we analyzed whether these animals developed heart complications. The heart damage was quantified by serum CK-MB activity, an enzyme located in the cytosol of the cardiomyocytes that is released into the blood after heart damage^19^. The activity of CK-MB was significantly increased in *T*. *cruzi*-infected *Pik3cg*^*−/−*^ mice compared with its activity in WT mice (Fig. 3a), indicating an enhancement of the heart damage in *Pik3cg*^*−/−*^ mice. *Pik3cg*^*KD/KD*^ mice also showed high levels of serum CK-MB after *T*. *cruzi* infection (Fig. 3b). Further supporting heart dysfunction in *Pik3cg*^*−/−*^ mice after infection, echocardiography revealed a concentric hypertrophy, along with a clear reduction in left ventricle diameter and cardiac output and no reduction in ejection fraction (Fig. 3c–g). These findings are indicative of a pathological adaptation of the heart leading to heart failure with preserved left ventricular ejection fraction^20,21^. Overall, the preceding data suggest that the canonical PI3Kγ signaling pathway protects mice from *T*. *cruzi* infection-induced heart damage and dysfunction.

### Chagasic myocarditis is enhanced in PI3Kγ-deficient mice

**Fig. 4:**
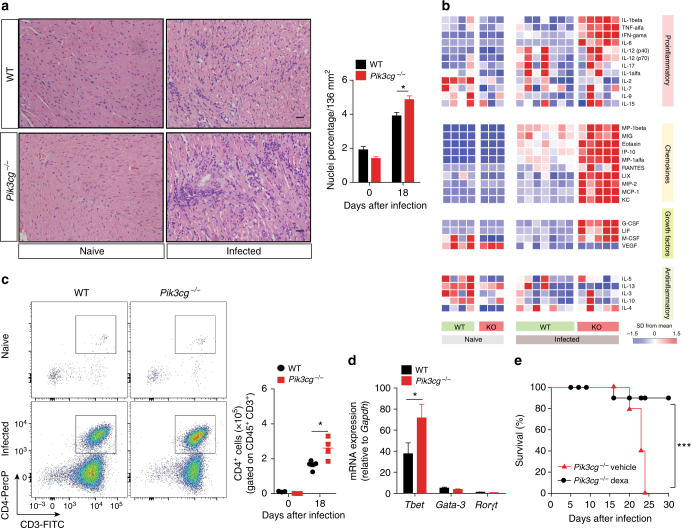
PI3Kγ controls the heart inflammation after infection with *T*. *cruzi*. **a** Representative images from H&E staining of heart tissue from non-infected WT (*n* = 6) and *Pik3cg*^*−/−*^ (*n* = 6) mice or 18 days after infection with 10^3^ trypomastigote forms of *T*. *cruzi* Y strain. Scale bar = 50 μm. **b** Heat map demonstrating the profile of cytokines and chemokines produced in the heart tissue of non-infected WT and *Pik3cg*^*−/−*^ mice or 18 days post infection with 10^3^ trypomastigote forms of *T*. *cruzi* Y strain. **c** Representative flow cytometry plots for the analysis of CD4 staining and quantification of the number of positive cells in the heart tissue of WT (*n* = 5) and *Pik3cg*^*−/−*^ (*n* = 4) naive mice or infected with *T*. *cruzi* Y strain. **d** PCR analysis of mRNA expression of the transcription factors *Tbet*, *Gata-3*, and *Rorγt* in the heart tissue of WT (*n* = 4) and *Pik3cg*^*−/−*^ (*n* = 4) mice. *Gapdh* was used as a housekeeping gene. **e** Survival rate of *Pik3cg*^*−/−*^ mice infected with 10^3^ trypomastigote forms of *T*. *cruzi* Y strain and treated at day 9 post infection with 2 mg Kg^−^ and at days 12 and 15 post infection with 1 mg Kg^−^ of dexamethasone (*n* = 5) or vehicle (*n* = 5). *P < 0.05 and ***P < 0.001 (one-way ANOVA with Tukey’s post hoc test in **a**, unpaired Student’s *t-*test in **c**, **d**, and Mantel–Cox log-rank test in **e**). Data are representative of two (**b**,** e**) or three (**a**, **c**, **d**) independent experiments (mean ± s.e.m in **a**, **c**, **d**)

To further understand why the canonical PI3Kγ signaling pathway protects mice from *T*. *cruzi*-induced heart damage and dysfunction, we next analyzed heart myocarditis in these mice. Although PI3Kγ signaling is very important for leukocyte migration in several models of inflammation^22–25^, histopathological analysis of the heart tissue from *T*. *cruzi*-infected *Pik3cg*^*−/−*^ mice revealed an increase in leukocytes infiltration in comparison to WT mice (Fig. 4a). In agreement with this finding, multiplex analyses of cytokines/chemokines profile in the heart tissue of *Pik3cg*^*−/−*^ (Fig. 4b and Supplementary Table 1) and *Pik3cg*^*KD/KD*^ (Supplementary Fig. 4 and Supplementary Table 2) mice revealed an increase in the levels of pro-inflammatory cytokines (e.g., TNF, IFN-γ, and IL-6), and chemokines (e.g., RANTES, MCP-1, and MIP-1α), whereas anti-inflammatory cytokines are downregulated (e.g., IL-10 and IL-13) when compared to infected WT mice. At the same period of infection, the production of pro-inflammatory and anti-inflammatory cytokines in the blood and spleen was not different between WT and *Pik3cg*^*−/−*^ mice (Supplementary Fig. 5), suggesting that the PI3Kγ signaling modulates inflammatory response in *T*. *cruzi* infection is specific for the heart tissue. Supporting the enhanced myocarditis, the number of CD4^+^ T-cells infiltrating the heart tissue of *Pik3cg*^*−/−*^-infected mice was increased compared to WT mice (Fig. 4c). Consistent with human chagasic cardiomyopathy^26^, the real-time qPCR analysis in the heart revealed that CD4^+^ T-cells mainly express the mRNA of the Th1-related transcription factor *Tbet*, compared with Th2 (*Gata3*) and Th17 (*Rorgt)* cells (Fig. 4d). In the absence of PI3Kγ, *Tbet* expression was increased compared to WT controls (Fig. 4d). In addition, post treatment of *Pik3cg*^*−/−*^-infected mice with a low dose of dexamethasone (glucocorticoid) reversed their susceptibility to *T*. *cruzi* infection (Fig. 4e). Collectively, these results indicate that enhanced myocarditis of infected *Pik3cg*^*−/−*^ and *Pik3cg*^*KD/KD*^ mice might explain enhanced heart dysfunction and mortality.

**Fig. 5:**
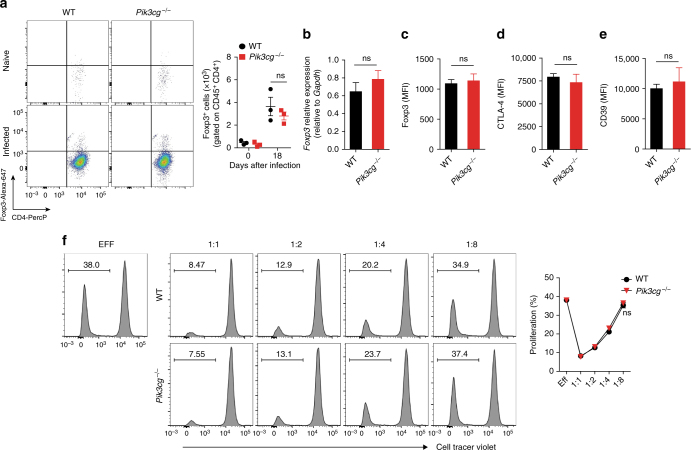
The absence of PI3Kγ does not affect the number and suppressive function of regulatory T-cells in the heart tissue of infected mice. **a** Representative flow cytometry plots for the analysis of Foxp3 staining and quantification of the number of positive cells in the heart tissue of WT (*n* = 4) and *Pik3cg*^*−/−*^ (*n* = 3) naive mice or infected with *T*. *cruzi* Y strain. **b** PCR analysis of mRNA expression of *Foxp3* gene in the heart tissue of WT (*n* = 4) and *Pik3cg*^*−/−*^ (*n* = 4) mice. *Gapdh* was used as a housekeeping gene. **c**–**e** Flow cytometry analysis measured by the mean fluorescence intensity (MFI) of the cell surface markers Foxp3 (**c**), CTLA-4 (**d**), and CD39 (**e**) expressed in CD4^+^Foxp3^+^ cells from the heart tissue of infected of WT (*n* = 4) and *Pik3cg*^*−/−*^ (*n* = 4) mice 18 days after infection with 10^3^ trypomastigote forms of *T*. *cruzi* Y strain. **f** In vitro T-cell suppression by Treg cells isolated from the lymph nodes of WT and *Pik3cg*^*−/−*^ naive mice. ns = no statistical significance (unpaired Student’s *t-*test in **a**–**f**). Data are from one experiment (**f**) or representative of two (**a**–**e**) independent experiments (mean ± s.e.m in **a**–**f**)

Since PI3Kγ signaling appears to controls the production of inflammatory mediators after infection with *T*. *cruzi*, we investigated whether this effect might be related to the number and suppressive function of regulatory T-cells. It was found that the number of CD4^+^ Foxp3^+^ regulatory T-cells (Tregs) was not different in the heart tissue of *Pik3cg*^*−/−*^ and WT mice at 18 dpi (Fig. 5a). Furthermore, the expression of the regulatory markers in Tregs from infected heart of WT and *Pik3cg*^*−/−*^, Foxp3 (Fig. 5b, c), CTLA-4 (Fig. 5d), and CD39 (Fig. 5e) were also similar. In addition, the PI3Kγ deficiency did not interfere with the suppressor function of Tregs (CD4^+^ CD25^+^) cells isolated from lymph nodes of naive mice (Fig. 5f). These results ruled out that a disturbance in Tregs cells might explain the enhanced chagasic myocarditis of *Pik3cg*^*−/−*^ mice compared to WT controls.

### PI3Kγ signaling in myeloid cells reduces *T*. *cruzi* infection

**Fig. 6:**
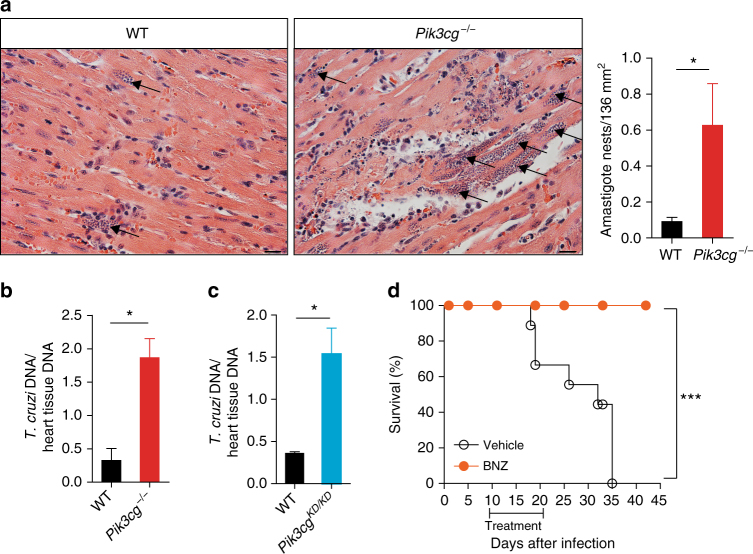
PI3Kγ controls the heart parasitism after infection with *T*. *cruzi*. **a** Representative images from H&E staining of heart tissue from WT (*n* = 6) and *Pik3cg*^*−/−*^ (*n* = 6) mice 18 days post infection with *T*. *cruzi* Y strain. Black arrows indicate amastigote nests of the parasite. Scale bars = 20 µm. **b**, **c** Quantitative PCR analysis of *T*. *cruzi* DNA isolated from *Pik3cg*^*−/−*^ (**b**) and *Pik3cg*^*KD/KD*^ (**c**) mice 18 days post infection with *T*. *cruzi* Y strain. **d** Survival rate of *Pik3cg*^*−/−*^ mice infected with 10^3^ trypomastigote forms of *T*. *cruzi* Y strain and treated with 100 mg Kg^−^ of benznidazole (BNZ) (*n* = 8) or vehicle (*n* = 6). *P < 0.05, by unpaired Student’s *t*-test, relative to WT mice (**a**–**c**). *P < 0.05 and ***P < 0.001 (unpaired Student’s *t*-test in **a**–**c** and Mantel–Cox log-rank test in **d**). Data are from one experiment (**d**) or representative of two (**c**) or three (**a**,** b**) independent experiments (mean ± s.e.m in **a**–**c**)

Because PI3Kγ signaling deficiency leads to a large number of inflammatory cells and higher levels of inflammatory mediators in the heart tissue of infected mice, we evaluated whether these cells were able to control heart parasitism. Of note, an increased number of amastigote nests were detected in the heart of *Pik3cg*^*−/−*^ mice when compared to WT mice (Fig. 6a). According to the histological findings, the heart tissue of infected *Pik3cg*^*−/−*^ or *Pik3cg*^*KD/KD*^ mice showed increased amount of parasite DNA compared to infected WT mice (Fig. 6b, c). Supporting the hypothesis that *Pik3cg*^*−/−*^ mice died due to uncontrolled heart infection, post treatment (after parasitemia has falling down) with benznidazole, the standard trypanocidal drug used for *T*. *cruzi* infection^27^, protected *Pik3cg*^*−/−*^ mice from death when compared to vehicle-treated mice (Fig. 6d).

Heart *T*. *cruzi* parasitism control is orchestrated mainly by infiltrating CD8^+^ T-cells and activated macrophages^28–31^. Thus, we evaluated the presence and function of these cells to elucidate the mechanisms of uncontrolled heart parasitism in *Pik3cg*^*−/−*^ mice. Numbers of infiltrating CD8^+^ T-cells in the heart of *Pik3cg*^*−/−*^-infected mice did not differ from WT mice (Supplementary Fig. 6a). In addition, the in vivo cytotoxic activity of CD8^+^ T-cells in infected *Pik3cg*^*−/−*^ mice was similar to WT mice (Supplementary Fig. 6b), indicating that a defect in CD8^+^ T-cells was not responsible for the *Pik3cg*^*−/−*^ mice susceptibility to control heart parasitism.

**Fig. 7:**
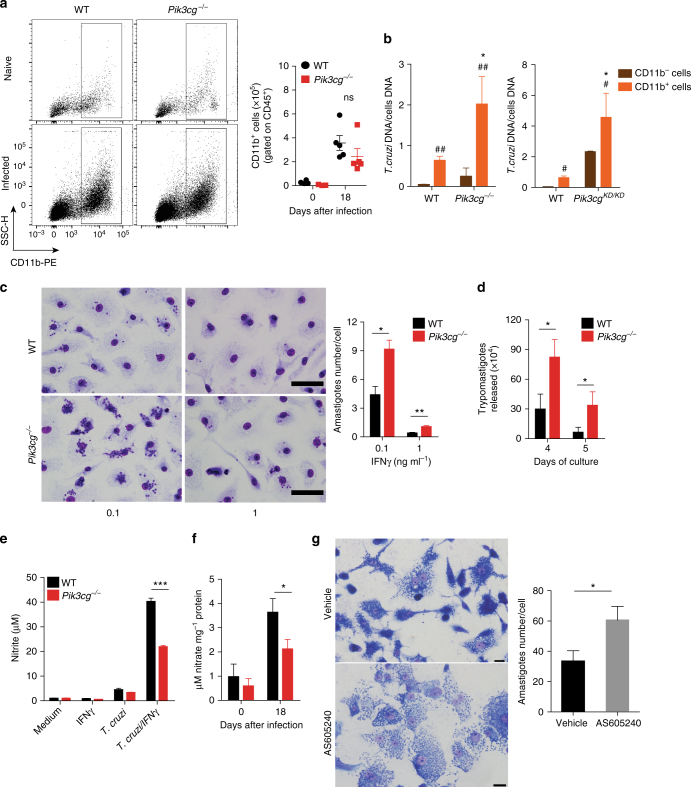
Disruption of PI3Kγ signaling in human or murine macrophages harms the killing of intracellular *T*. *cruzi*. **a** Representative flow cytometry dot plots for the analysis of CD11b staining and quantification of the absolute number of positive cells in the heart tissue of non-infected WT (*n* = 5) and *Pik3cg*^*−/−*^ (*n* = 5) mice or 18 days post infection with 10^3^ trypomastigote forms of *T*. *cruzi* Y strain. **b** Quantitative PCR analysis of nanogram of *T*. *cruzi* DNA presents in 1 ng of DNA from CD11b^−^ and CD11b^+^ cells isolated from the heart tissue of WT and *Pik3cg*^*−/−*^ or *Pik3cg*^*KD/KD*^-infected mice. **c** Representative images of BMDMs stimulated with 0.1 or 1 ng of IFN-γ and infected for 48 h with trypomastigote forms of *T*. *cruzi* Y strain at MOI 3:1. Scale bars = 100 µm. **d** Trypomastigote forms of *T*. *cruzi* Y strain released from culture of WT and *Pik3cg*^*−/−*^ BMDM stimulated with 0.1 ng ml^−^ of IFN-γ. Experiments were performed in triplicate. **e** Nitrite quantification by Griess reaction in the supernatant of WT and *Pik3cg*^*−/−*^ BMDMs stimulated with 1 ng of IFN-γ and infected with *T*. *cruzi* Y strain at MOI 3:1. **f** Nitrate quantification in the heart tissue of WT (*n* = 7) and *Pik3cg*^*−/−*^ (*n* = 7) naive mice or 18 days post infection with 10^3^ trypomastigote forms of *T*. *cruzi* Y strain. **g** Representative images of human macrophage lineage THP-1 cells treated with specific PI3Kγ inhibitor AS605240 (1 µM) and infected with trypomastigote forms of *T*. *cruzi* Y strain at MOI 3:1. Scale bars = 20 µm. ns = no statistical significance (unpaired Student’s *t*-test in **a**). *P < 0.05 relative to WT CD11b^+^ cells and ^#^*P* < 0.05 and ^##^*P* < 0.01 relative to WT or *Pik3cg*^*−/−*^ CD11b^−^ cells (unpaired Student’s *t*-test in **b**). *P < 0.05, **P < 0.01, and ***P < 0.001 (unpaired Student’s *t*-test **c**–**g**). Data are one experiment (**g**) or representative of two (**a**–**d**, **f**) or three (**e**) independent experiments with similar results (mean ± s.e.m in **a**–**g**)

To further investigate why *Pik3cg*^*−/−*^ mice failed to control cardiac parasitism, we next determined whether a dysfunction in migration and/or function of myeloid cells (e.g., macrophages) could be involved. First, recruitment of CD11b^+^ cells into the heart of infected *Pik3cg*^*−/−*^ mice was similar to infected WT mice (Fig. 7a). However, CD11b^+^ cells isolated from the heart of *Pik3cg*^*−/−*^ or *Pik3cg*^*KD/KD*^ mice exhibited higher parasite levels compared with CD11b^+^ cells isolated from WT mice (Fig. 7b). Although the parasitism in CD11b^−^ cells from *Pik3cg*^*−/−*^ and *Pik3cg*^*KD/KD*^ mice was also higher, intracellular parasite levels were lower than in CD11b^+^ cells (Fig. 7b). To further understand the mechanisms by which *Pik3cg*^*−/−*^-deficient myeloid cells failed to control intracellular *T*. *cruzi* infection, we investigated the ability of bone marrow-derived macrophages (BMDMs) from *Pik3cg*^*−/−*^ mice to control the intracellular *T*. *cruzi* in vitro.

Corroborating the in vivo findings, BMDMs from *Pik3cg*^*−/−*^ mice treated with IFN-γ were remarkably less effective in killing the parasite than WT BMDMs (Fig. 7c). In addition, the number of trypomastigote forms released into the supernatant from the *Pik3cg*^*−/−*^ BMDM culture was higher than in supernatant from the WT BMDM culture (Fig. 7d). The increase in the nitric oxide (NO) production by macrophages is the most important mechanism involved in their microbicidal activity against *T*. *cruzi*^32,33^. In association with the impaired ability to kill intracellular *T*. *cruzi*, infected *Pik3cg*^*−/−*^ BMDMs produced lower amounts of NO compared to infected WT BMDMs (Fig. 7e). We then proceeded to investigate whether the heart macrophages from *Pik3cg*^*−/−*^ mice had a reduced parasite-killing capacity because they were not able to produce NO in vivo. PI3Kγ was found to be essential for NO production in the heart of *T*. *cruzi*-infected mice since the concentration of nitrate in the infected heart tissue was reduced in *Pik3cg*^*−/−*^ mice as compared with the WT group (Fig. 7f). Then, we investigated whether PI3Kγ signaling was also important for the microbicidal function of human macrophages against *T*. *cruzi* infection in vitro. Corroborating the mouse data, pharmacological inhibition of PI3Kγ reduced the ability of human macrophages (THP-1) to kill intracellular parasites (Fig. 7g).

**Fig. 8:**
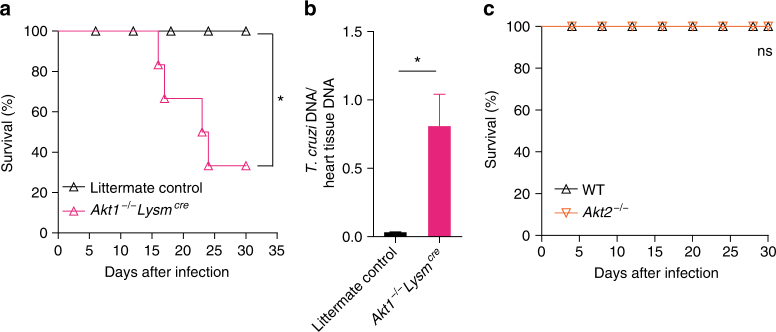
AKT1 signaling in myeloid cells confers resistance to *T*. *cruzi* infection. **a** Survival rate of littermate control (*n* = 7) and *Akt1*^*−/−*^*Lysm*^*cre*^ (*n* = 7) mice after infection with 10^3^ trypomastigote forms of *T*. *cruzi* Y strain. **b** Quantitative PCR analysis of picogram of *T*. *cruzi* DNA presents in 1 ng of heart tissue DNA isolated from WT (*n* = 5) and *Akt1*^*−/−*^*Lysm*^*cre*^ (*n* = 5) mice. **c** Survival rate of WT (*n* = 7) and AKT2^−/−^ (*n* = 7) mice infected with 10^3^ trypomastigote forms of *T*. *cruzi* Y strain. *P < 0.05 and ns = no statistical significance (Mantel–Cox log-rank test in **a**, **c** and unpaired Student’s *t*-test in **b**). Data are representative of two independent experiments (mean ± s.e.m in **b**)

Finally, to validate that the canonical PI3Kγ signaling in myeloid cells was important to counteract heart parasitism and confer resistance to infection in vivo, mice harboring a conditional deletion of AKT1 in LysM^+^ cells (*Akt1*^*−/−*^*Lysm*^*cre*^) were used^34^. Of note, *Akt1*^*−/−*^*Lysm*^*cre*^ mice were more susceptible to *T*. *cruzi* infection when compared to control littermates (Fig. 8a). In agreement, heart parasitism was not controlled in *Akt1*^*−/−*^*Lysm*^*cre*^ mice (Fig. 8b). Conversely, *Akt2*^*−/−*^ mice were as resistant to *T*. *cruzi* infection as WT mice (Fig. 8c), indicating that AKT1, but not AKT2, was involved in the downstream events of PI3Kγ signaling responsible for the control of *T*. *cruzi* infection. Taken together, these results indicated that a canonical PI3Kγ/AKT1 signaling deficiency in myeloid cells led to an inability to control heart *T*. *cruzi* infection and ultimately avoid heart damage and host death.

### PI3Kγ signaling in human chagasic myocarditis

**Fig. 9:**
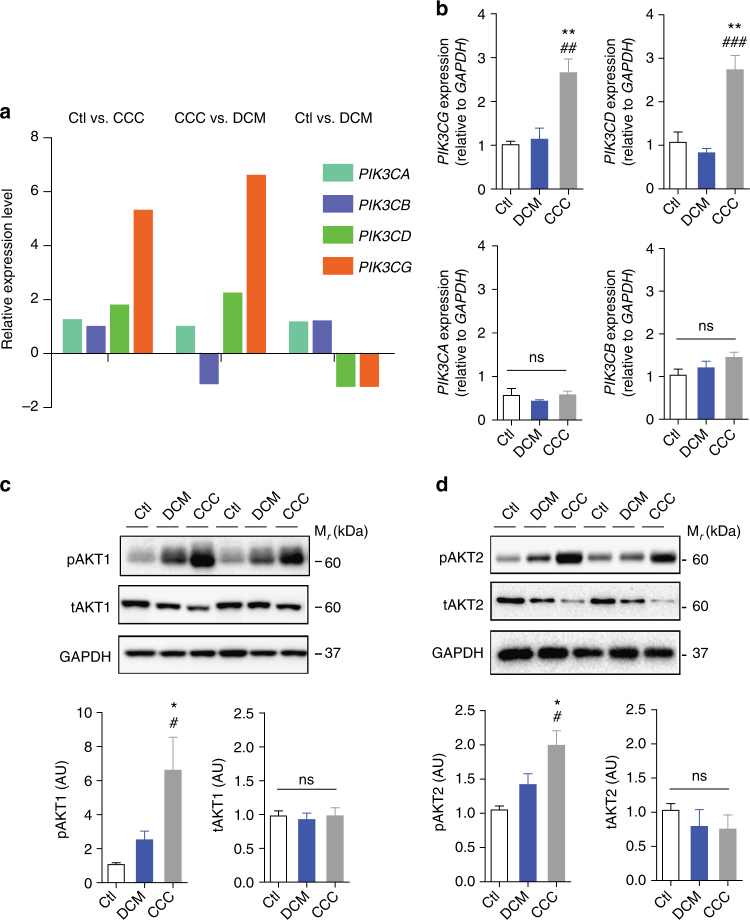
The PI3Kγ signaling is enhanced in the human heart tissue with Chagas disease. **a** Transcriptome analysis of family I *PIK3* genes expression (*PIK3CA*, *PIK3CB*, *PIK3CD*, and *PIK3CG*) in healthy control (Ctl; *n* = 7) and patients with idiopathic dilated cardiomyopathy (DCM; *n* = 14) or chronic chagasic cardiomyopathy (CCC; *n* = 10). **b** RT-PCR analysis of the mRNA expression of *PIK3CA*, *PIK3CB*, *PIK3CD*, and *PIK3CG* genes in the heart tissue of Ctl (*n* = 5); DCM (*n* = 10); and CCC (*n* = 10) patients. *GAPDH* was used as a housekeeping gene. **c** Representative western blots and analysis of phosphorylated (p) and total (t) AKT1 expression in the heart tissue of Ctl (*n* = 5), DCM (*n* = 10), and CCC (*n* = 10) patients. GAPDH was used as a loading control. **d** Representative western blots and analysis of phosphorylated (p) and total (t) AKT2 expression in the heart tissue of Ctl (*n* = 5), DCM (*n* = 10), and CCC (*n* = 10) patients. GAPDH was used as a loading control. AU refers to arbitrary units. ns = no statistical significance (one-way ANOVA with Tukey’s post hoc test in **b**–**d**). *P < 0.05 and **P < 0.01 relative to Ctl and #*P* < 0.05, ##*P* < 0.01, and ###*P* < 0.001 relative to DCM (one-way ANOVA with Tukey’s post hoc test in **b**–**d**). Data are from one experiment (mean ± s.e.m in **b**–**d**)

To translate our findings in the mouse model to the human disease, we examined the expression and activation of PI3Kγ signaling in the heart samples of chagasic disease patients. Human heart tissue transcriptome (GSE84796) revealed that the expression of *PIK3CG* gene was upregulated in the samples from patients with CCC when compared to heart samples from healthy control individuals (Ctl) (relative expression = 5.37; *P* = 1.0E-5; Student’s *t-*test with the Benjamini–Hochberg method) or even samples from patients with non-chagasic cardiomyopathy (idiopathic dilated cardiomyopathy, DCM, GSE111544, Relative expression = 6.60; *P* = 2.1E-8; Student’s *t-*test with the Benjamini–Hochberg method) (Fig. 9a). None of the other members of the class 1 PI3K family (α, β, and δ subunits) appeared to be modulated as dramatically as the *PIK3CG* gene (Fig. 9a). To validate the transcriptome data, samples from human hearts were used for the evaluation of *PIK3CG* expression by real-time PCR. Corroborating our transcriptome analyses, *PIK3CG* and *PIK3CD* expression were upregulated (Fig. 9b), whereas the expression of *PIK3CA* and *PIK3CB* was similar in the heart samples from CCC patients in comparison to those from healthy control individuals or DCM patients (Fig. 9b). Next, we verified the activation of PI3Kγ signaling in the chagasic human samples. Although there was no change in *AKT1* and *AKT2* mRNA (GSE84796 and validation—Supplementary Fig. 1c, d) and protein expression (Fig. 9c, d), the activated forms of AKT1 and AKT2 (pAKT1 and pAKT2) were increased in the heart tissue from CCC patients compared to those from DCM patients or healthy controls (Fig. 9c, d). To analyze the interactions between PI3Kγ and the heart immune response, a correlation analysis was performed using the transcriptome data set from CCC patients (GSE84796). The expression of the *PIK3CG* gene positively correlated with the expression of myeloid cells markers (CD11b, CD300a, and CD33) and inflammatory mediators genes (Supplementary Fig. 7a–h), indicating that the increased influx of leukocytes in chagasic hearts might be the cause of the increase in PI3Kγ expression. To validate these findings, real-time PCR was performed in the heart samples. The expression of pro-inflammatory cytokines (*TNF* and *IFNG*) and chemokines (*CCL5/RANTES*) was higher in heart tissue of CCC patients compared with the DCM or control groups (Supplementary Fig. 8a–c). In agreement, there was a positive correlation in heart tissue of CCC patients between the expression of the *PIK3CG* gene and the proinflammatory genes *TNF* (*r*^2^ = 0.42; *P* = 0.16, Pearson’s test), *IFNG* (*r*^2^ = 0.62; *P* = 0.061, Pearson’s test), and *CCL5/RANTES* (*r*^2^ = 0.68; *P* = 0.042, Pearson’s test) (Supplementary Fig. 8d–f).

**Fig. 10:**
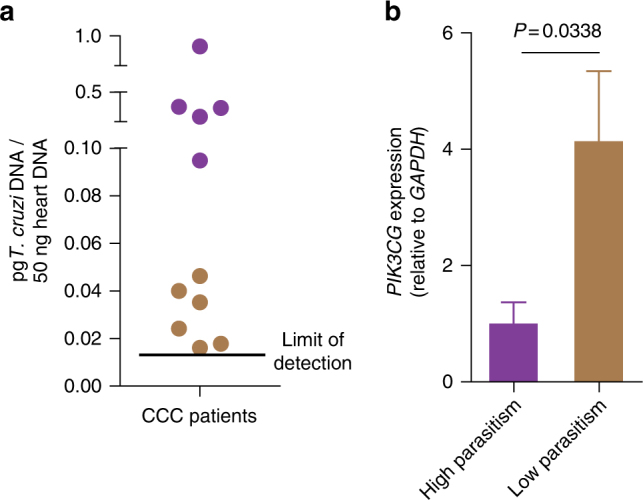
Relationship between parasitism and PI3Kγ expression in the heart tissue of CCC patients. **a** Quantitative real-time PCR analysis of *T*. *cruzi* DNA levels (pg/50 ng of human DNA) in heart tissue from CCC patients. The black line indicates the lowest amount of parasite detected by reaction. **b** qRT-PCR analysis of the mRNA expression of *PIK3CG* in the heart tissue of CCC patients segregated by high (*n* = 5) or low (*n* = 6) heart parasitism. Unpaired Student’s *t-*test in **b**. Data are from one experiment (mean ± s.e.m in **b**)

Finally, we also analyzed the interaction between PI3Kγ expression and heart parasitism to validate the hypothesis that PI3Kγ participates in the mechanisms that control heart *T*. *cruzi* infection. Real-time PCR analyses of *T*. *cruzi* DNA in the heart samples of CCC patients revealed two distinct groups of patients regarding the levels of parasitism. Some of them had low levels of heart parasitism, which were almost undetectable, and others showed significant parasitism (Fig. 10a). When we stratified these two groups, we found that the expression of *PIK3CG* was much higher in the group with low parasitism (Fig. 10b). Collectively, these results indicated that similarly the findings obtained in the mouse model of *T*. *cruzi* infection, PI3Kγ signaling was also upregulated in the heart tissue of one group of chagasic disease patients and that this signaling might also be acting as a counteracting mechanism of heart *T*. *cruzi* parasitism.

## Discussion

Characterization of the pathophysiological aspects of heart *T*. *cruzi* infection and the mechanisms that lead to resistance or susceptibility of the host is fundamental to understand the Chagas disease and to propose novel targets to prevent the development of the severe form of this disease. In this regard, identification of the intracellular signaling pathways activated in response to *T*. *cruzi* infection is critical. In the present study, we investigated the role of PI3Kγ signaling in the heart of *T*. *cruzi*-infected mice and humans. Our findings indicate that canonical PI3Kγ/AKT1 signaling is upregulated in the heart tissue of *T*. *cruzi*-infected mice and humans with the Chagas disease. Finally, we further demonstrated an essential role of the canonical PI3Kγ signaling in myeloid cells (e.g., macrophages) to restrict heart parasitism and consequently avoid heart inflammation/damage and dysfunction.

The activation of PI3K signaling has been demonstrated during in vitro infections of human and mouse macrophages with *T*. *cruzi*^35–37^. To our knowledge, this is the first study to provide further evidence that canonical PI3Kγ signaling, together with its downstream pathway AKT1, is activated in the heart tissue from chagasic patients and mice infected with *T cruzi*. Herein, we found that in the absence of canonical PI3Kγ signaling in hematopoietic cells, mice are highly susceptible to *T*. *cruzi* infection, which is associated with an uncontrolled heart parasitism and exacerbated inflammatory response. Since PI3Kγ pathway is activated by GPCRs, such as chemokine receptors, this signaling is crucial for leukocyte migration, genetic or pharmacological inhibition of this kinase decreases the inflammation and ameliorates the score in several models of inflammatory diseases^22–24,38^. Our data showed that the ablation of PI3Kγ does not inhibit the *T*. *cruzi*-induced leukocyte migration to the heart tissue, suggesting that during *T cruzi* infection, leukocyte migration is not mediated by canonical PI3Kγ signaling, and other chemotaxis signaling events might play a role during the infection.

It is established that PI3Kγ signaling also plays an important role in cardiomyocytes physiology and that its absence can aggravate some cardiomyopathies^18,39^. Our data indicate that the participation of PI3Kγ signaling in the pathophysiology of chagasic heart disease seems to be more related to its function in heart-infiltrating myeloid cells. This result is apparently different from the evidence showing that during the chronic phase of *T*. *cruzi* infection, the PI3K/AKT/NO axis in cardiomyocytes contributes to the development of electric dysfunction of cardiomiocytes^40^. Nevertheless, these differences could be explained by the different stage of the disease or even by different PI3K isoform involved^40^. Furthermore, the impact of the PI3K/AKT/NO pathway in cardiomyocytes on the in vivo outcome has been not evaluated^40^.

Chagasic heart failure has been attributed to increased levels of local or systemic pro-inflammatory cytokines/chemokines^27^. In addition to the inability to restrict the heart parasitism, our data showed an enhanced production of pro-inflammatory cytokines and chemokines in the cardiac tissue of *Pik3cg*^*−/−*^ mice. Although this “cytokine storm” might be merely a consequence of uncontrolled parasitism, it seems strictly important for the increased susceptibility. This hypothesis was supported by the findings that treatment with a low dose of glucocorticoid is able to reverse *Pik3cg*^*−/−*^ mouse susceptibility. Nevertheless, we cannot eliminate the possibility that PI3Kγ signaling pathway directly dampen the inflammatory response in the heart of infected mice. In fact, a recent study has demonstrated that the activation of PI3Kγ signaling counteracts inflammatory cytokines and chemokines production by macrophages^41^.

The finding that canonical PI3Kγ signaling is also increased in the heart tissue of CCC patients is consistent with the observed tissue leukocyte infiltration. Furthermore, our finding that patients with lower heart parasitism have higher cardiac expression of PI3Kγ supports our hypothesis that this kinase contributes to the containment of *T*. *cruzi* infection. On the other hand, some patients develop CCC despite having high expression/activation of canonical PI3Kγ signaling and controlling the parasitism in the heart. Nevertheless, as myocarditis is the prime process of CCC progression^42^, partial control of heart parasitism due to PI3Kγ-expressing myeloid cells occurs at the expense of tissue-damaging inflammation, likely leading to myocarditis and the development of cardiomyopathy in such chronically infected individuals. Consistently, we propose that PI3Kγ signaling might be a protective mechanism that restricts heart parasitism to avoid a rapid progression of the disease, which usually takes several years to chronify. Finally, we hypothesize that alterations that change PI3Kγ expression and/or function (due to polymorphisms, mutations) could be a factor involved in the susceptibility of some patients to developing cardiac forms of Chagas disease. Further studies are necessary to test this hypothesis.

Collectively, the present study has demonstrated the crucial role of the canonical PI3Kγ signaling for host resistance to *T*. *cruzi* infection. Furthermore, the canonical PI3Kγ signaling in heart-infiltrating myeloid cells is crucial for optimal microbicidal activity that leads to the control of heart parasitism and to the avoidance of exacerbated myocarditis and heart damage. In conclusion, we believe that our data provided further insight into the molecular mechanisms of the pathophysiology of chagasic heart disease.

## Methods

### Animals

Female C57BL/6 WT, PI3Kγ-deficient mice (*Pik3cg*^*−/−*^)^43^, and PI3Kγ *kinase dead* mutant mice (*Pik3cg*^*KD/KD*^)^44^ aged 6–8 weeks were bred and maintained at the animal facility of the Ribeirão Preto Medical School University of São Paulo, Ribeirão Preto, Brazil. Female conditional *Akt1*^*−/−*^*Lysm*^*cre*^ were generated by crossing *Akt1*^*fl/fl*^ mice^34^ (kindly provided by Prof. P. Tsichlis, Tufts University, Boston, USA) and *Lysm*^*cre*^ mice (Jackson Laboratories). *Akt1*^*−/−*^*Lysm*^*cre*^ mice, littermate controls and *Akt2*^*−/−*^ mice^45^ (kindly provided by Prof. M. Birnbaum, U. Pennsylvania, Philadelphia, USA) were maintained at the animal facility of the Medical School, University of Crete and IMBB-FORTH, Greece. *Pik3cg*^*+/*−^ mice were generated by crossing *Pik3cg*^*−/−*^ mice with C57BL/6 WT mice. The animals were maintained in micro-isolator cages under standard conditions and fed with food and water ad libitum. The Institutional Ethics Committee in Animal Experimentation—CEUA of the Ribeirão Preto Medical School, University of São Paulo (Protocol number 196/2009) and the Veterinary Department of the Crete Region, Medical School, University of Crete and IMBB-FORTH, Heraklion, Crete, Greece (Protocol number 27298/2014) approved the experimental protocols. Mice were selected based on their genotype. All other criteria were not considered and as such, randomized. We were not blinded to the mouse genotypes or group allocations except where mentioned.

### Mouse infection

All experiments were conducted using trypomastigote forms of human isolated *T*. *cruzi* Y strain^46^. For in vitro experiments, parasites were grown for 7–9 days in a fibroblast cell line LLC-MK_2_ (American Type Culture Collection-ATCC®, cat. CCL-7™) and suspended in RPMI 1640 medium (Gibco-BRL Life Technologies, Grand Island, NY, USA). For in vivo experiments, mice were inoculated intraperitoneally with 10^3^ bloodstream trypomastigote forms. From day 5 after infection, blood parasitemia was evaluated in 5 μl of blood from the tail vein by counting 100 fields through direct observation under an optical microscope. Additionally, the body weight of the mice was also measured. The survival rate was assessed daily. Infected animals were excluded (pre-established) from the study when parasitemia was not detected at day 9 post infection.

### Real-time quantitative PCR

For RT-PCR, total RNA was isolated form heart samples with TRIzol using the specific kit RNeasy mini kit (Qiagen), and cDNA was synthesized with the ThermoScript RT-PCR system (Invitrogen). PCR was carried with StepOnePlus (Applied Biosystems) using SYBR Green GoTaq qPCR MasterMix (Promega). The endogenous control *Gapdh* was used to normalize gene expression. The sequences of the primers are shown in Supplementary Table 3.

### Western blot analysis

Protein lysates from human or mouse heart samples were prepared in RIPA buffer supplemented with a cocktail of protease and phosphatase inhibitors (Cell Signaling). Equal amounts of total proteins were loaded and separated in SDS-polyacrylamide gel. After electrophoresis, the proteins were electrotransferred onto nitrocellulose membranes (0.2 μm, GE Healthcare) and incubated for 1 h with 5% bovine serum albumin followed by overnight incubation at 4 °C with primary antibody. Finally, the membranes were incubated for 1 h with secondary antibodies conjugated to horseradish peroxidase. Immunoblots were visualized with the Western Blotting Chemiluminescence Luminol (Pierce, Thermo Fisher Scientific Inc.) using the ChemiDoc™ XRS System (Bio-Rad). The primary antibodies used in the study were: phospho-AKT1 (1:600; Cell Signaling, cat. 9018); total AKT1 (1:1000; Cell Signaling, cat. 2938); phospho AKT2 (1:600, Cell Signaling, cat. 8599); total AKT2 (1:1000; Cell Signaling, cat. 3063); and GAPDH (1: 5000; Sigma-Aldrich, cat. G9545). Full scans of the original uncropped western blots are shown in Supplementary Figs. 9 and 10.

### Serum CK-MB activity assay

To evaluate cardiac damage, the activity of CK-MB was measured in the sera of mice infected with *T*. *cruzi*. Quantification was performed using a kinetic method with specific kit CK-MB Liquiform (Labtest®) followed by read in spectrophotometer at 340 nm (EMAX Molecular Devices Corporation®).

### Echocardiography

Mice were anesthetized with 1.5% isofluorane (Forane® Isoflurane, USP) in an inhalation chamber. Body temperature was monitored, and cardiac parameters were obtained through VEVO2100® machine using a 30 MHz transducer. Diastolic left ventricle diameter and ventricular posterior wall thickness were evaluated in M mode; ejection fraction and cardiac output were calculated in bidimensional mode.

### Histological analysis

Heart samples were fixed in 10% buffered formalin solution and, after 72 h, dehydrated in a crescent concentration of ethanol solutions and embedded in paraffin. Blocks were sectioned at thickness of 5 μm and stained with haematoxylin-eosin (H&E). Using a light microscope, the number of amastigote nests was counted in 25 images of each animal of the group, the inflammation was estimated with ImageTool 2.0 software (University of Texas Health Science Center, TX, USA). The pathologist who performed the histological analysis was blinded to the mice genotypes.

### Treatment with PI3Kγ inhibitor

WT mice were divided in two groups and treated subcutaneously with 30 mg kg^−^ of PI3Kγ inhibitor AS605240 (5-Quinoxalin-6-ylmethylene-thiazolidine-2,4-dione—Alexis®) or vehicle (2% DMSO)^47^. Mice were treated once a day starting from the peak of parasitemia (day 9 of infection) for 10 days.

### Cytokines and chemokines measurement

Measurement of cytokines and chemokines in the heart tissue of mice was performed by Milliplex® (Merck Millipore) assay in accordance with the manufacturer’s instructions.

Production of the cytokines IFN-γ, TNF-α, IL-10, and IL-4 (R&D Systems, Minneapolis, MN) was assessed in the serum and spleen of WT and *Pik3cg*^*−/−*^ naive mice or 18 days after infection with 10^3^ trypomastigote forms of *T*. *cruzi* Y strain. Fragments of spleen were collected into vials with a protease inhibitor cocktail (Complete, Roche), macerated, and centrifuged, and the supernatant collected for cytokine quantification. Measurements were performed through by ELISA assay, using specific kit (DuoSet®, R&D Systems) according to manufacturer’s instructions.

### FACS analysis

The mouse heart tissue was sliced into small pieces and digested using Liberase TL (Roche) for 1 h at 37 °C/5% CO_2_. The obtained cells suspension was filtered through a 40 µm cell strainer (Corning®), and isolated cells were counted in a Neubauer chamber and then incubated with specific antibodies. The following antibodies were used: APC-Cy7 rat anti-mouse CD45 (1: 200, clone 30-F11, BD Biosciences, cat. 557659); FITC armenian hamster anti-mouse CD3 (1:250, clone 145-2C11, eBioscience, cat. 11-0031-63); PercP rat anti-mouse CD4 (1:250, clone RM4-5, BD Biosciences, cat. 553052); PE rat anti-mouse CD8a (1:250, clone 53-6.7, BD Biosciences, cat. 553033); Alexa Fluor® 647 rat anti-mouse Foxp3 (1:100, clone R16-715, BD Biosciences, cat. 563486); PE rat anti-CD11b (1:250, clone M1/70, BD Biosciences cat. 553311); PE hamster anti-mouse CD152/CTLA-4 Clone (1:150, UC10-4F10-11, BD Bioscience, cat. 553720); PE/Cy7 anti-mouse CD39 (1:250, clone Duha59 BioLegend, cat. 143806). For each sample, a minimum of 1 million events were acquired using a BD FACSVerse cytometer driven by the FACSuite software (BD Biosciences, San Diego, EUA). The obtained data were analyzed using the software FlowJo version X (Tree Star, Inc.). Gating strategies for flow cytometry analysis are schematically represented in Supplementary Fig. 11.

### Heart CD11b^+^ and CD11b^−^ cells isolation

The mouse heart tissue was sliced in small pieces and digested using Liberase TL (Roche) for 1 h at 37 °C/5% CO_2_. The obtained cell suspension was filtered through a 40-µm cell strainer (Corning®). Isolated cells were stained with PE rat anti-CD11b (1:300; clone M1-70, BD Biosciences, cat. 553311) and then incubated with anti-PE magnetic beads (Miltenyi Biotec). The cell suspension was separated into CD11b^+^ and CD11b^−^ samples using MACS® cell separation columns (Miltenyi Biotec) according to the manufacturer’s instructions.

### Total genomic DNA extraction and parasitism analysis

Total genomic DNA was extracted from 10 mg of CCC patients or mouse heart tissue or from mouse heart CD11b^+^ and CD11b^−^ cells using a specific kit (Wizard® SV Genomic DNA Purification System Promega, cat. A2361) according to the manufacturer’s instructions. Real-time PCR was performed to quantify the heart parasitism as previously described^48^.

### Treatment with dexamethasone

WT and *Pik3cg*^*−/−*^ mice were infected with 10^3^ trypomastigote forms of *T*. *cruzi* Y strain and treated intraperitoneally at day 9 post infection with 2 mg Kg^−^ and at days 12 and 15 with 1 mg Kg^−^ of dexamethasone or vehicle.

### Treatment with benznidazole

*Pik3cg*^*−/−*^ mice were infected with 10^3^ trypomastigote forms of *T*. *cruzi* Y strain and treated by gavage with 100 mg Kg^−^ of Benznidazole (BNZ) or vehicle. Mice were treated once a day starting from the day 9 post infection for 10 days.

### BM cells isolation and generation of chimeric mice

WT and *Pik3cg*^*−/−*^ BM stem cells were obtained by flushing the femur. Briefly, the bone was surgically removed, and muscle tissue was scraped away from bone using a scissor and scalpel blade. Subsequently, the epiphysis was cut, and the stem cells were removed from the diaphysis by flushing with RPMI 1640 medium using a syringe with a 26-gauge needle. Cells were centrifuged at 500 × *g* 10 min at 4 °C, suspended in PBS, counted using trypan blue solution (Sigma-Aldrich®) and diluted at 2.5 × 10^7^ ml^−^ (5 × 10^6^/200 µl) for intravenous injection. To generate the chimeras, 10–12-weeks-old WT and *Pik3cg*^*−/−*^ mice were irradiated using a Cesium 137 source irradiator (Mark I model 25) at 9 Gy. On the day after irradiation, the mice were divided at the following groups: (1) WT mice repopulated with WT BM cells; (2) *Pik3cg*^*−/−*^ mice repopulated with WT BM cells; and (3) *Pik3cg*^*−/−*^ mice repopulated with *Pik3cg*^*−/−*^ BM cells. After BM transplantation, the mice were treated for 15 days with the antibiotic Ciprofloxacin hydrochloride diluted in drinking water at 10 mg ml^−^. After 2 months (period required for BM engraftment), mice were infected with 500 trypomastigote forms of *T*. *cruzi* Y strain, and the survival rate was followed every day for 30 days.

### Differentiation of BMDM and killing assay

BMDMs were obtained as previously described^49^ and used for the killing activity assay, release of *T*. *cruzi* assay or NO quantification after stimulation with IFNγ and *T*. *cruzi*. For the killing activity assay, BMDMs (1.4 × 10^6^ ml^−^) were incubated overnight with different concentrations of IFNγ (0.1 and 1 ng ml^−^) and macrophages posteriorly were infected with *T*. *cruzi* at a MOI 3:1 ratio. After 4 h, the cells were washed to remove non-internalized parasites and incubated for an additional 48 h at 37 °C a 5% CO_2_. The macrophage killing activity was evaluated by counting of intracellular amastigote forms internalized per 150 cells. The counts were performed in a blinded manner. For the release of *T*. *cruzi* assay, 2 × 10^5^ cells were incubated with 1 ng ml^−^ of IFNγ and after 12 h were infected with *T*. *cruzi* at a MOI of 3:1. At days 4 and 5 post infection trypomastigote forms of the parasites released to the medium were counted^50^.

### THP-1 cells differentiation and killing activity assay

Authenticated THP-1 cells (cat. TIB-202, ATCC®, Manassas, VA, USA) monocytes lineage was differentiated into macrophages using the method previously described by Daigneault et al^51^. Briefly, THP-1 cells were cultured with 200 nM of PMA (Sigma-Aldrich; Merck Millipore) for 3 h at 37 °C, 5% CO_2_. After incubation, PMA-containing medium was removed, cells were plated in 24-wells plate at 2 × 10^5^/well and the cells were incubated for an additional 3 days. At the third day, THP-1 were treated with 1 µM of PI3Kγ inhibitor AS605240 and infected with *T*. *cruzi* at a MOI 3:1 ratio followed by an additional 48 h of incubation at 37 °C in a 5% CO_2_ incubator. After 48 h, the cells were fixed and stained with Panótico (Laborclin®). The macrophage killing activity was evaluated by counting (blinded) of intracellular amastigote forms internalized per 50 cells. These cells were not contaminated with mycoplasma.

### Nitrite and nitrate quantification

The nitrite concentration in the culture supernatants of BMDM was determined using the conventional Griess reaction method^52^. For the nitrate quantification assay, heart samples removed near the papillary muscle were homogenized with a sonicator (Virsonic 100, Virtis, NY, USA) in 200 μl of cold 0.1 N acetic acid, centrifuged for 5 min (9300 *g*) and the supernatant used for measurement of nitrate and total protein by the Bradford method. Subsequently, 25 µl of supernatant was deproteinized with 50 µl of cold ethanol and maintained at −20 °C for 30 min. Then, the samples were centrifuged (9300 *g*, 5 min) and the nitrate content was determined using a chemiluminescent analyzer (Sievers 280 NO Analyzer, Boulder, CO, USA). The values are expressed in µM nitrate per μg of protein.

### In vivo cytotoxicity assay of CD8^+^ cells

Eighteen dpi, WT and *Pik3cg*^*−/−*^ mice intravenously received two populations of splenocytes from WT naive mice previously stained with two different concentrations of carboxyflourescein (CFSE—Sigma Aldrich®). The first population was stained with CFSE at 10 μM (CFSE high) and pulsed for 40 min with the *T*. *cruzi* peptide PA8, which is presented based on class I MHC as the target of CD8^+^ cells. The second population stained with CFSE at 1 μM (CFSE low) and not pulsed with any peptide was used as the control population. Recipient WT and *Pik3cg*^*−/−*^ mice were sacrificed approximately 16 h after the cell transfer, and splenocytes were analyzed with a flow cytometer to determine the percentage of CFSE high and low populations.

### CD4^+^CD25^+^ suppression assay

CD4^+^ T-cells were isolated by negative selection from lymph nodes of WT mice using CD4 MACS beads Multisort (Miltenyi Biotec), according to the manufacturer’s recommendations. Briefly, the non-CD4^+^ T-cells were labeled with a mix containing cocktail of biotin-conjugated antibodies and anti-biotin monoclonal antibodies conjugated to microbeads. The magnetically labeled cells were subsequently depleted by separation using AutoMacs Pro Separator Cell sorter (Miltenyi Biotec). Next, CD4^+^ cells obtained were stained with PE anti-mouse CD25 (1:200, clone PC61.5, eBioscience, cat. 12-0251-81) and the CD25^+^ PE-labeled cells were incubated with anti-PE microBeads and isolated by positive selection. Two distinct cell populations, CD4^+^CD25^−^ (effectors T-cells) and CD4^+^CD25^+^ (Tregs) were obtained. Tregs were cultured with T effectors cells in a 96-well U-bottom plate (Costar®). The Teff cells were labeled with 1 μM Dye Efluor 670 (eBioscience) for 15 min at 37 °C, washed, and then cultured (1 × 10^5^ cells per well) for 4 days with a graded number of Tregs (Teff:Treg: 1:1, 1:2, 1:4, and 1:8) in the presence of soluble Purified hamster anti-mouse CD3e (3 μg/ml, Clone 145-2C11, BD Bioscience, cat. 553057) and Purified hamster anti-mouse CD28 (1.5 μg/mL, Clone 37.51, BD Bioscience, cat. 553294). Proliferation of Teff cells was determined by dye dilution in FACS analysis. The results are expressed as the percentage of suppression under each Treg:Teff ratio using the following formula: [proliferation of Teff only–(proliferation of Teff + Treg)/proliferation of Teff only] × 100.

### Patient samples and tissue collection

Experiments on human samples were performed in accordance with the ethical standards established in the Declaration of Helsinki. The protocol was approved by the Institutional Review Board of the School of Medicine of São Paulo (CAPPesq-protocol number 0265-10) and the Brazilian National Ethics in Research Commission (CONEP protocol number 009/2011). Written informed consent was obtained from the patients. Tissue specimens from the left ventricle free wall were obtained from individuals with end-stage heart failure at the moment of heart transplantation (DCM patients, age 41.1 ± 15.63 years; mean ± SD). All patients with CCC (age 46.1 ± 9.33 years; mean ± SD) were considered serologically positive for antibodies against *T*. *cruzi* based on the results of at least two of three independent tests. They underwent standard electrocardiography and echocardiography. They presented with typical electrocardiography findings and had severe ventricular dysfunction, with a left ventricular ejection fraction of <0.4. Similarly, human samples were obtained from patients with idiopathic DCM characterized by severe ventricular dysfunction, an absence of ischemic disease, and negative results of serologic tests for *T*. *cruzi* infection. Finally, control biopsy specimens were obtained from healthy hearts of organ donors (mean age 23 ± 4.69 years) for which there was no suitable recipient. Human experiments were blinded with respect to the samples group.

### Whole-transcriptome analysis

Whole-genome expression was performed with the SurePrint G3 Human Gene Expression 8 × 60K arrays (Agilent Technologies, Les Ulis, France), according to the manufacturer’s recommendations. Gene expression data were deposited in the GEO database (GSE84796 and GSE111544). Microarray analyses and signal normalization were performed with GeneSpring software (11.5.1, Agilent). For each sample, the *P-*value was obtained using the Student’s *t-*test and adjusted for the false discovery rate with the Benjamini–Hochberg method. Genes were considered differentially expressed if adjusted *P-*values were <0.05 and showed an absolute fold change >2.0.

### Statistical analysis

Results are expressed as the mean ± s.e.m. for in vitro and in vivo experiments. The normal distribution of data was analyzed by D’Agostino and Pearson test. The data set obtained by experiments with mice or primary cells assumed a normal distribution. Differences were considered to be statistically significant with *P* < 0.05 by unpaired Student’s *t*-test or ANOVA followed by Bonferroni post test. Survival curves were evaluated using the log-rank/Mantel–Cox test. For analysis of the correlation, Pearson’s test was carried out. All statistical analyses were performed using GraphPad Prism Software 5 except for the analysis of correlation, for which SPSS was used. No statistical methods were used to predetermine the sample size. Variation within each data set obtained by experiments with mice or primary cells was assumed to be similar between genotypes since all strains were generated and maintained on the same pure inbred background (C57BL/6).

### Data availability

The data supporting the findings of this study are available within the article and its Supplementary Information files or from the corresponding authors on reasonable request. RNA expression data are available at the Gene Expression Omnibus repository (GSE84796); (GSE111544); and (GSE41089).

## Electronic supplementary material

Supplementary Information
