# Supplementary material for: Canonical PI3Kγ signaling in myeloid cells restricts Trypanosoma cruzi infection and dampens chagasic myocarditis
Source: Nat Commun. 2018 Apr 17;9:1513. doi: 10.1038/s41467-018-03986-3 (PMC5904108; doi:10.1038/s41467-018-03986-3)
Supplement: Supplementary file 1 — Supplementary Information [file 41467_2018_3986_MOESM1_ESM.pdf]

Supplementary Information for

Canonical PI3Kγ signaling in myeloid cells restricts *Trypanosoma cruzi* infection and dampens chagasic myocarditis

Silva *et al.*,

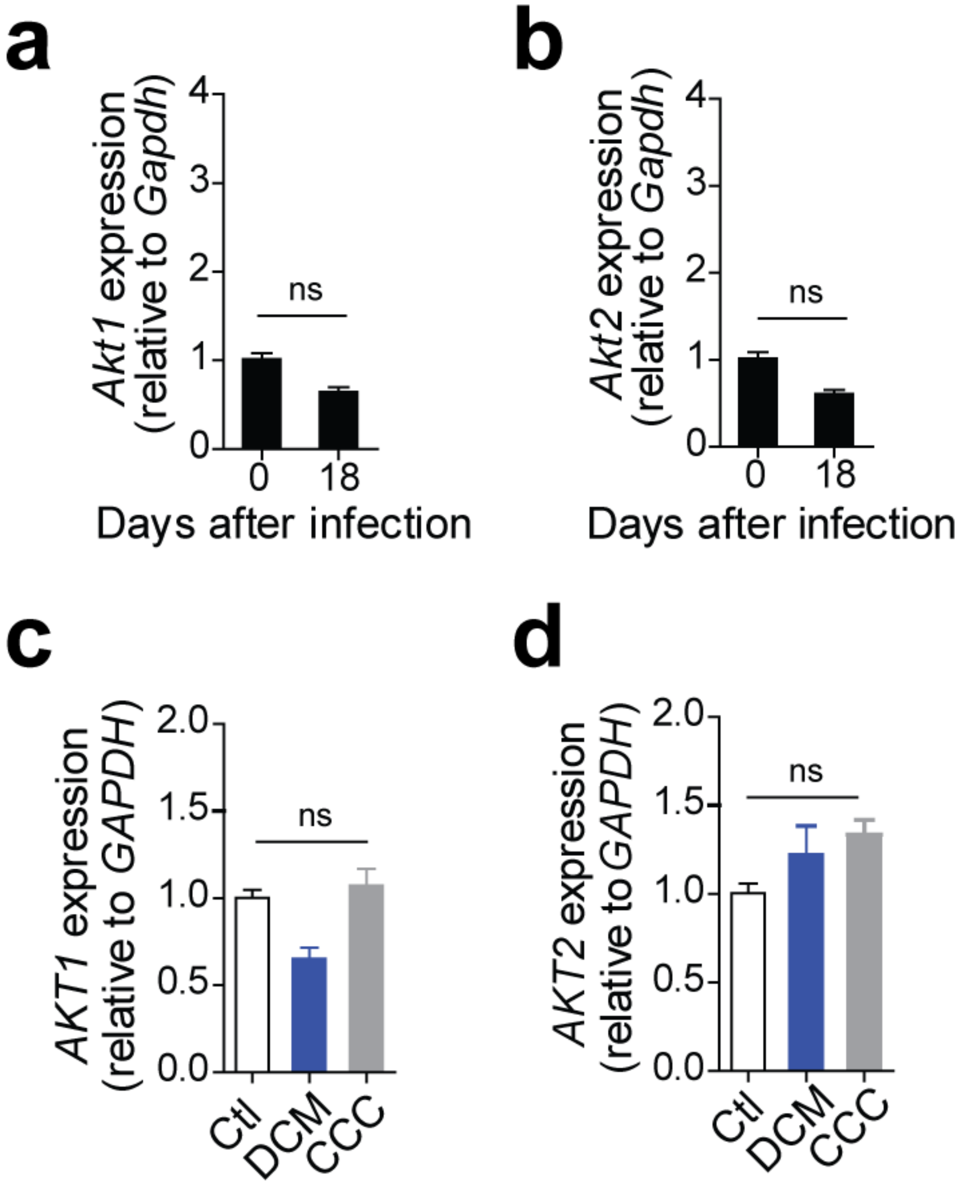

Supplementary Figure 1. AKT1 and AKT2 expression in the heart tissue of mice and humans infected with *T. cruzi*. (**a**-**b**) RT-PCR analysis of the mRNA expression of *Akt1* (**a**) and *Akt2* (**b**) genes in the heart tissue of C57BL/6 non-infected mice (n=7) or 18 days post infection with *T. cruzi* Y strain (n=11). (**c**-**d**) RT-PCR analysis of the mRNA expression of *AKT1* (**c**) and *AKT2* (**d**) genes in the heart tissue of Ctl (n=5); DCM (n=10) and CCC (n=10) patients. *Gapdh/GAPDH* was used as a housekeeping gene. ns=no statistical significance (**a**-**d**). (Unpaired Student's *t*-test in **a**,**b** and one-way ANOVA with Tukey's post-hoc test in **c**,**d**). Data are from one experiment (**c**,**d**) or representative of two (**a,b**) independent experiments (mean ± s.e.m in **a**-**d**).

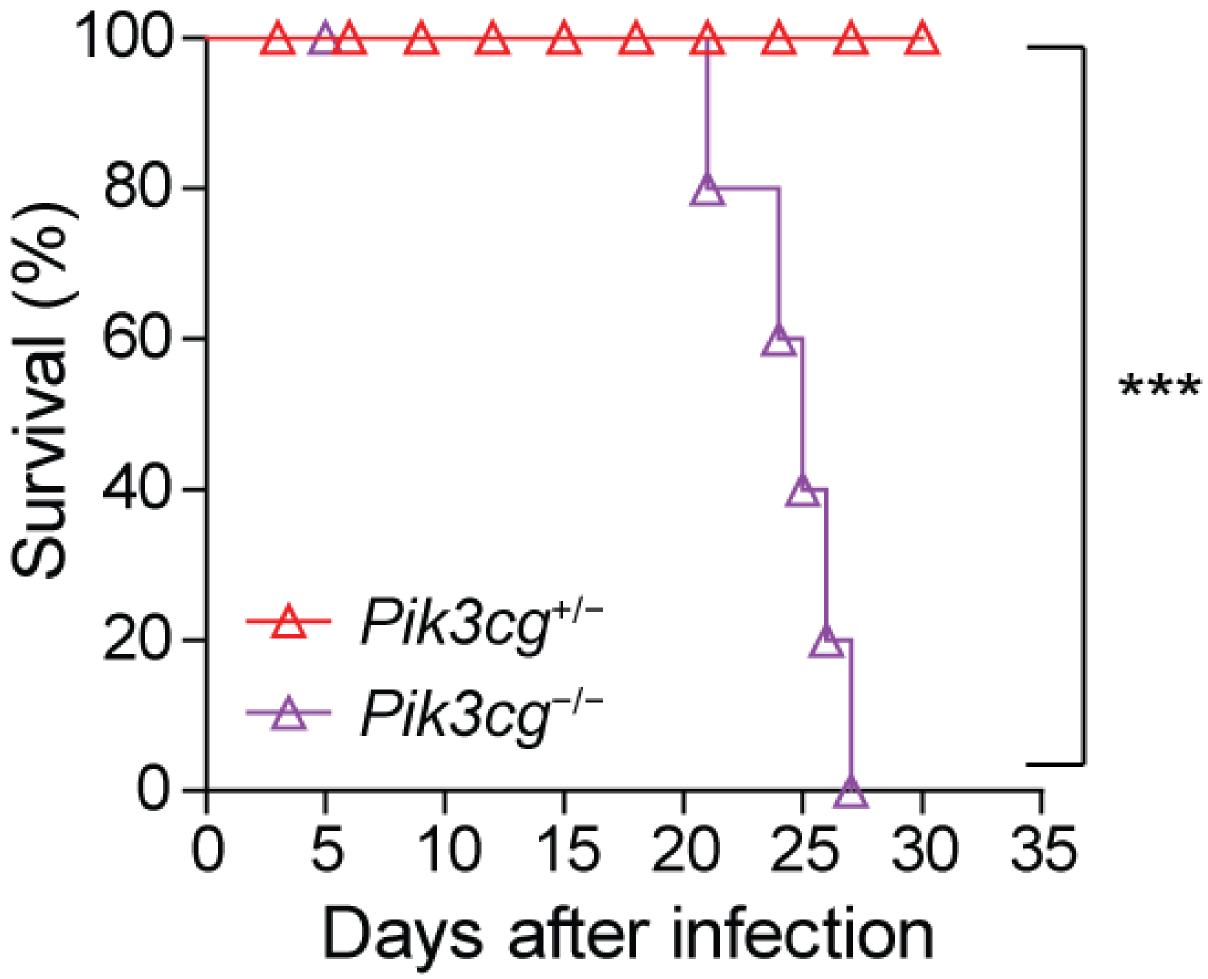

Supplementary Figure 2. Susceptibility to *T. cruzi* infection of *Pik3cg*$^{-/-}$ and *Pik3cg*$^{+/-}$ mice. Survival rate of homozygous *Pik3cg*$^{-/-}$ (n=5) and heterozygous *Pik3cg*$^{+/-}$ (n=10) infected with 10^3 trypomastigote forms of *T. cruzi* Y strain. ***P<0.001 (Mantel-Cox log-rank test). Data are representative of two independent experiments.

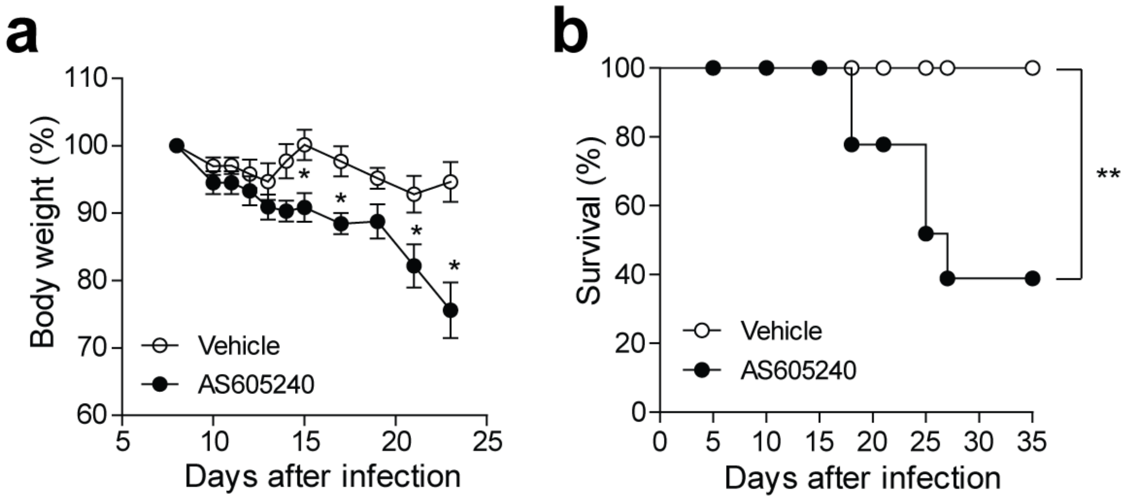

Supplementary Figure 3. Susceptibility of *T. cruzi* infected C57BL/6 mice treated with PI3Kγ inhibitor. Body weight (**a**) and survival rate (**b**) of WT mice treated with the selective PI3Kγ inhibitor AS605240 (n=8) or with vehicle (n=8). *P<0.05 and **P<0.01 (Student's *t* test in **a** and Mantel-Cox log-rank test in **b**). Data are representative of two (**a**,**b**) independent experiments (mean ± s.e.m in **a**).

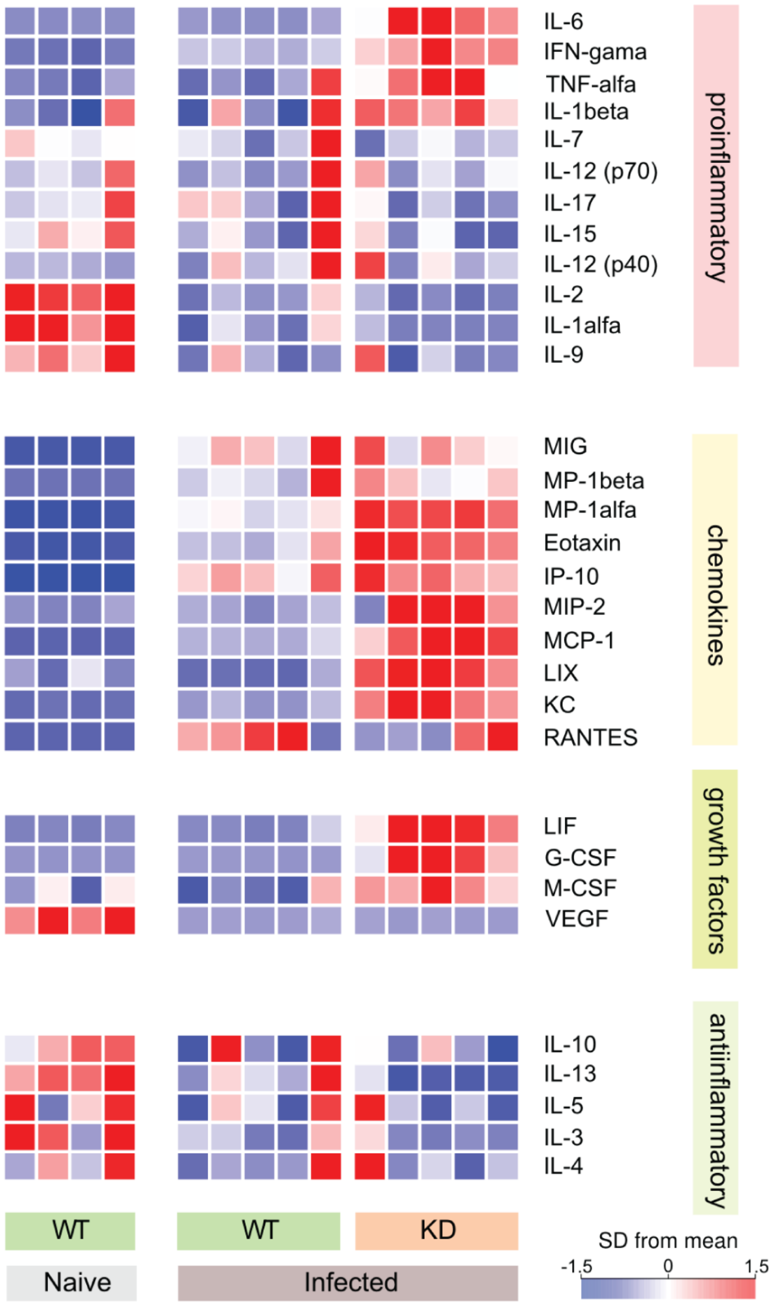

Supplementary Figure 4. Heat map related to cytokines and chemokines measurement in the heart of infected WT and *Pik3cg*$^{KD/KD}$ mice. Multiplex analysis of cytokines and chemokines produced in the heart tissue of WT and *Pik3cg*$^{KD/KD}$ mice before (n=4) and after (n=5) infection with 10^3 trypomastigote forms of *T. cruzi* Y strain. Data are from one experiment.

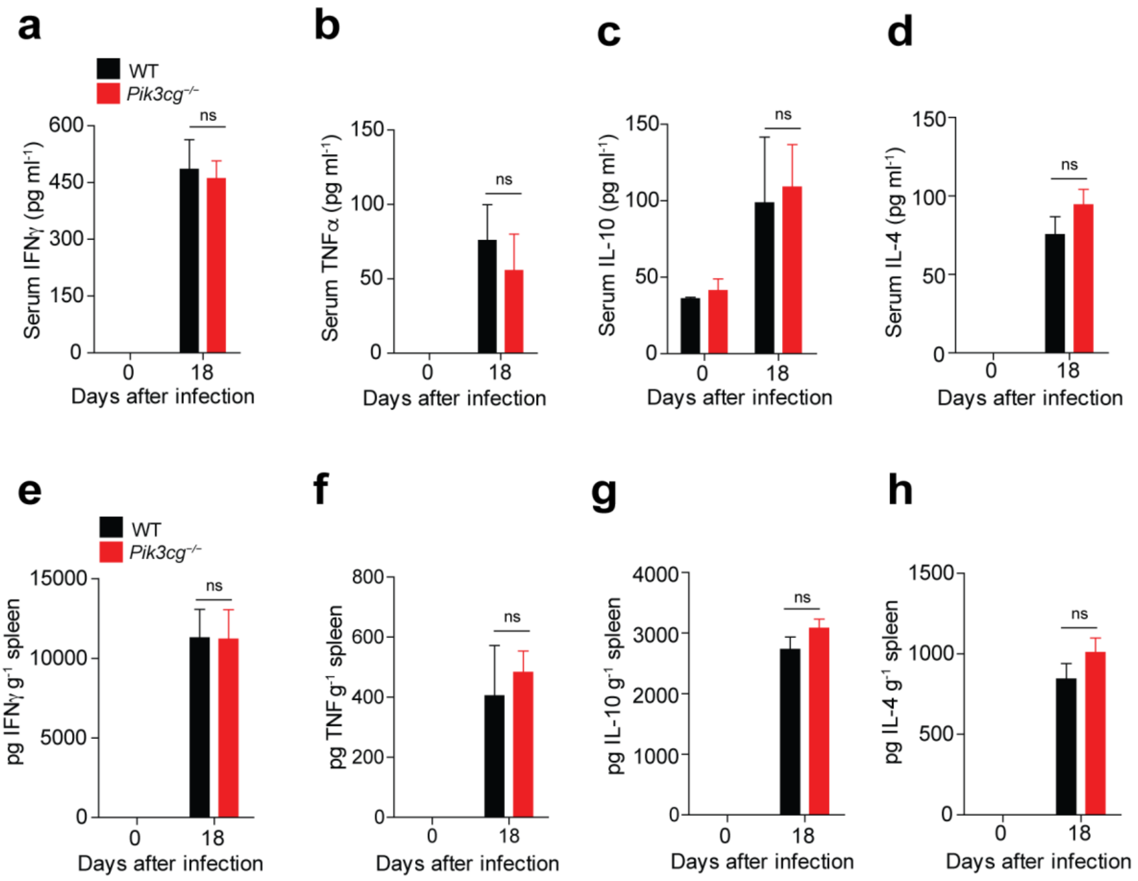

Supplementary Figure 5. Cytokine measurements in the serum and spleen of *Pik3cg*$^{-/-}$ mice after *T. cruzi* infection. ELISA assay to measure the levels of IFN-γ, TNF-α, IL-10 and IL-4 cytokines in the serum (**a-d**) and spleen (**e-h**) of WT (n=5) and *Pik3cg*$^{-/-}$ (n=6) naïve mice or 18 days post infection with 10^3 trypomastigote forms of *T. cruzi* Y strain. ns = no statistical significance (unpaired Student's *t*-test in **a-h**). Data are representative of two (**a-h**) independent experiments (mean ± s.e.m in **a-h**).

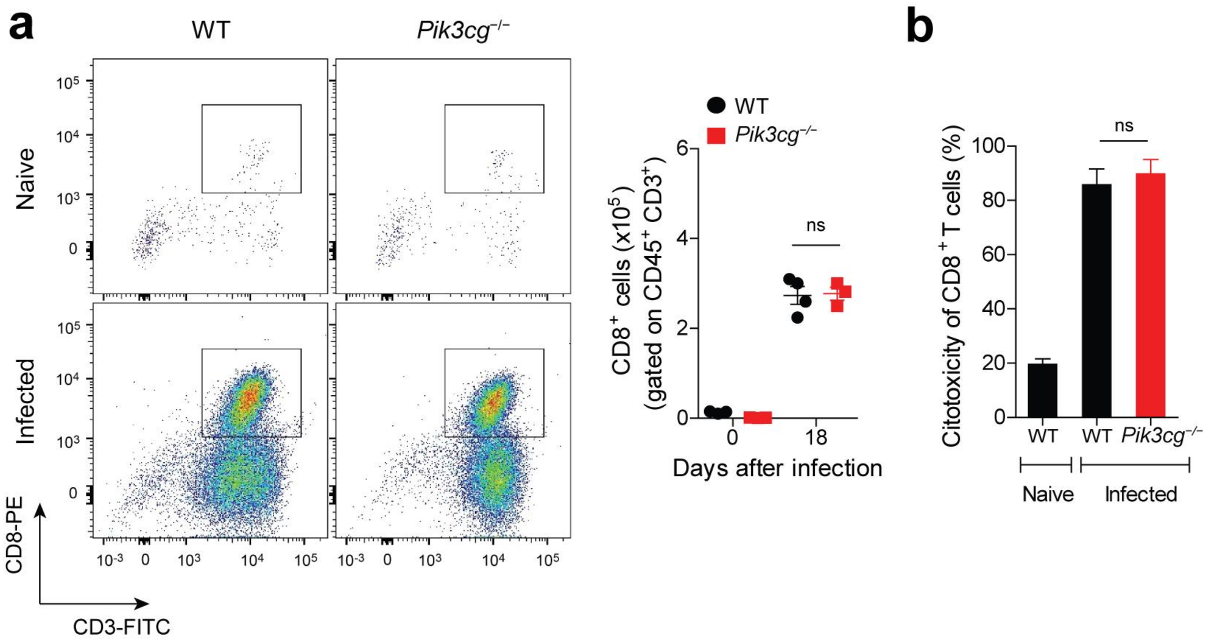

Supplementary Figure 6. Profile of CD8$^+$ T-cell infiltration in the heart tissue of WT and *Pik3cg*$^{-/-}$ infected mice. (**a**) Representative flow cytometry plots for the analysis of CD8 staining and quantification of the number of positive cells in the heart tissue of WT (n=5) and *Pik3cg*$^{-/-}$ (n=4) naïve mice or infected with 10^3 trypomastigote forms of *T. cruzi* Y strain. (**b**) *In vivo* assay of cytotoxicity of CD8$^+$ cells from WT (n=4) and *Pik3cg*$^{-/-}$ (n=4) mice infected with *T. cruzi*. ns=no statistical significance (unpaired Student's *t*-test in **a**-**b**). Data are representative of two (**b**) or three (**a**) independent experiments (mean ± s.e.m in **a**-**b**).

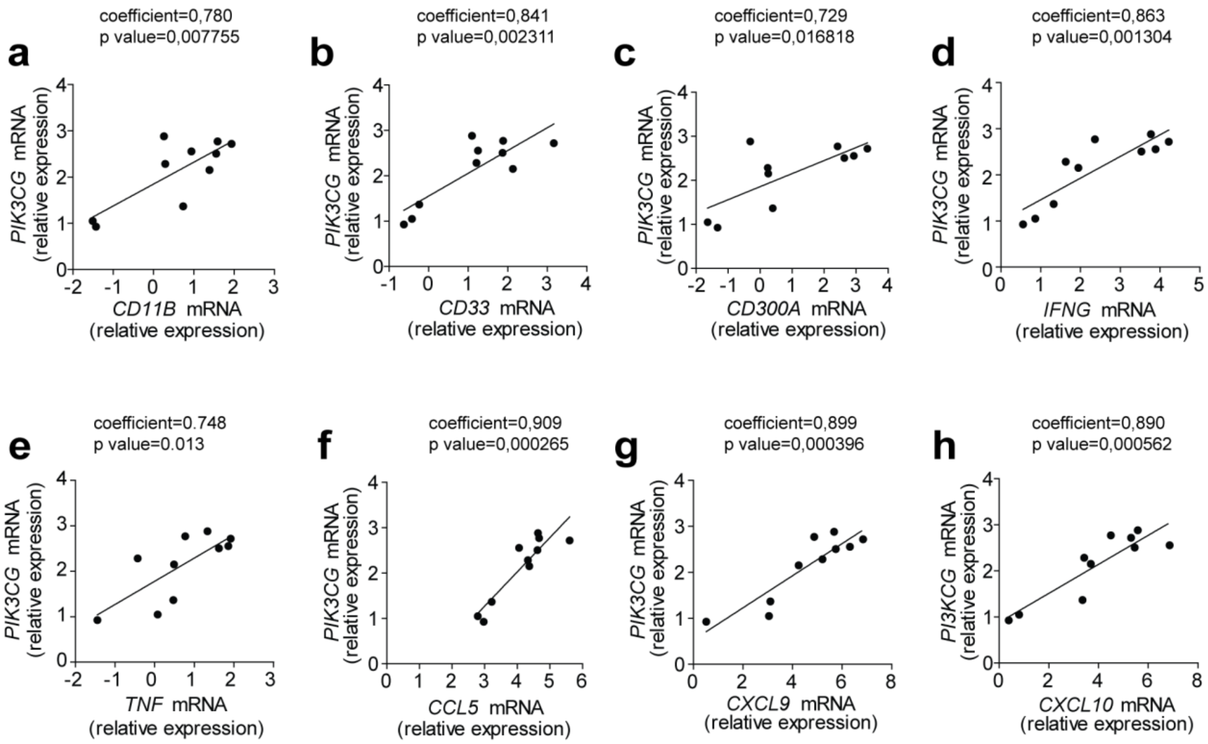

Supplementary Figure 7. Correlation between PI3Kγ relative expression and markers of inflammation in the heart tissue of CCC patients. (**a-h**) The transcriptome data set was used to perform a Spearman correlation analysis between the relative expression of the *PIK3CG* gene and several markers of inflammation in the heart tissue of CCC (n=10) patients. Pearson correlation coefficients and *P* values are shown in the panels.

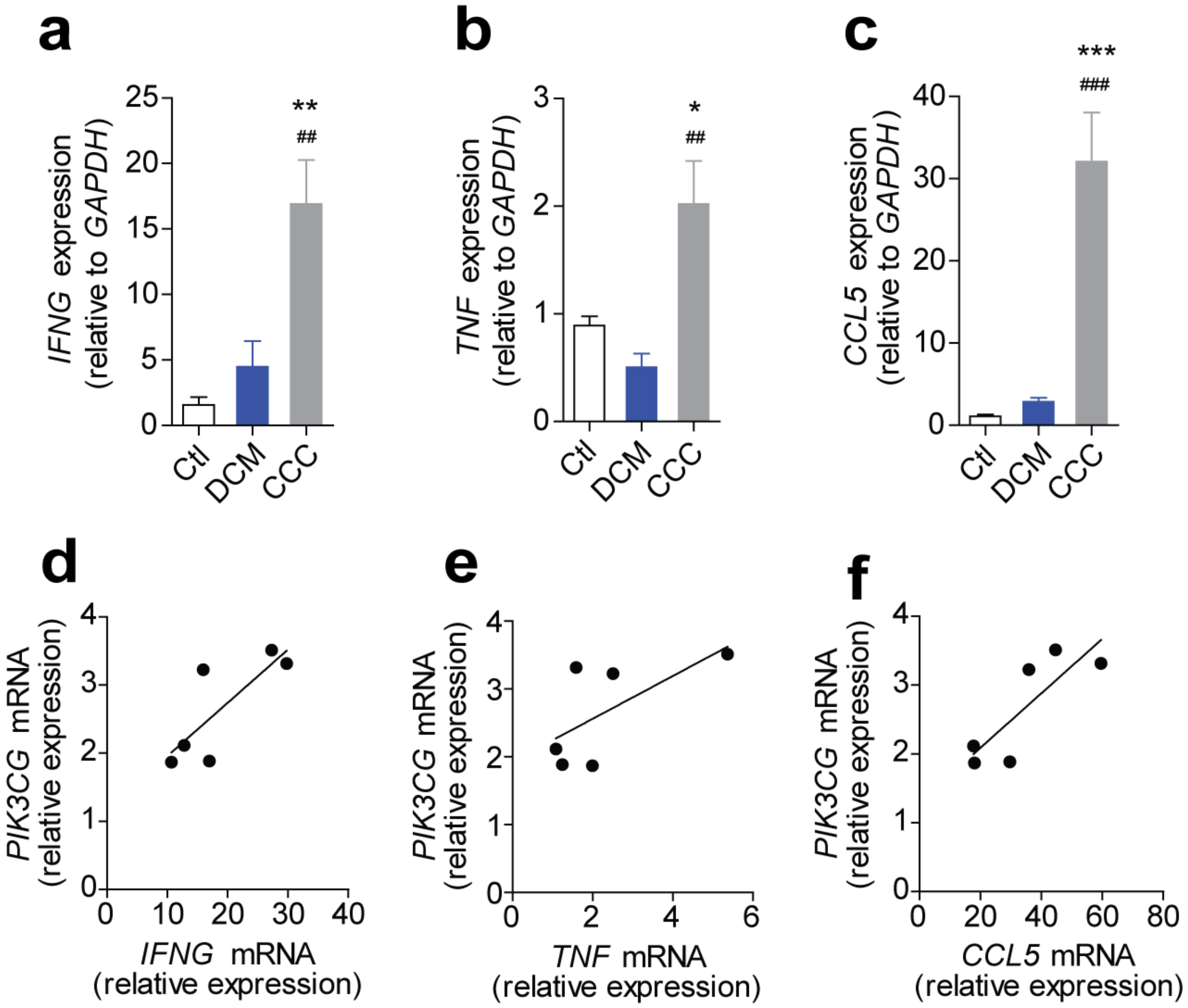

Supplementary Figure 8. Cytokines/chemokines expression and correlation with PI3Kγ expression in the heart tissue of CCC patients. (**a**-**c**) RT-PCR analysis of the mRNA expression of (**a**) *IFNG*, (**b**) *TNF*, and (**c**) *CCL5/RANTES* genes in the heart tissue of Ctl (n=5); DCM (n=6) and CCC (n=6) patients. *GAPDH* was used as a housekeeping gene. **P*<0.05, ***P*<0.01 and ****P*<0.001 relative to Ctl and ##*P*<0.01 and ###*P*<0.001 relative to DCM (one-way ANOVA with Tukey's post-hoc test in **a**-**c**). (**d**-**f**) Pearson correlation analysis between the gene expression of cytokines/chemokines and *PIK3CG* in the heart tissue of CCC (n=6) patients. Data are from one experiment in **a**-**f** (mean ± s.e.m in **a**-**c**).

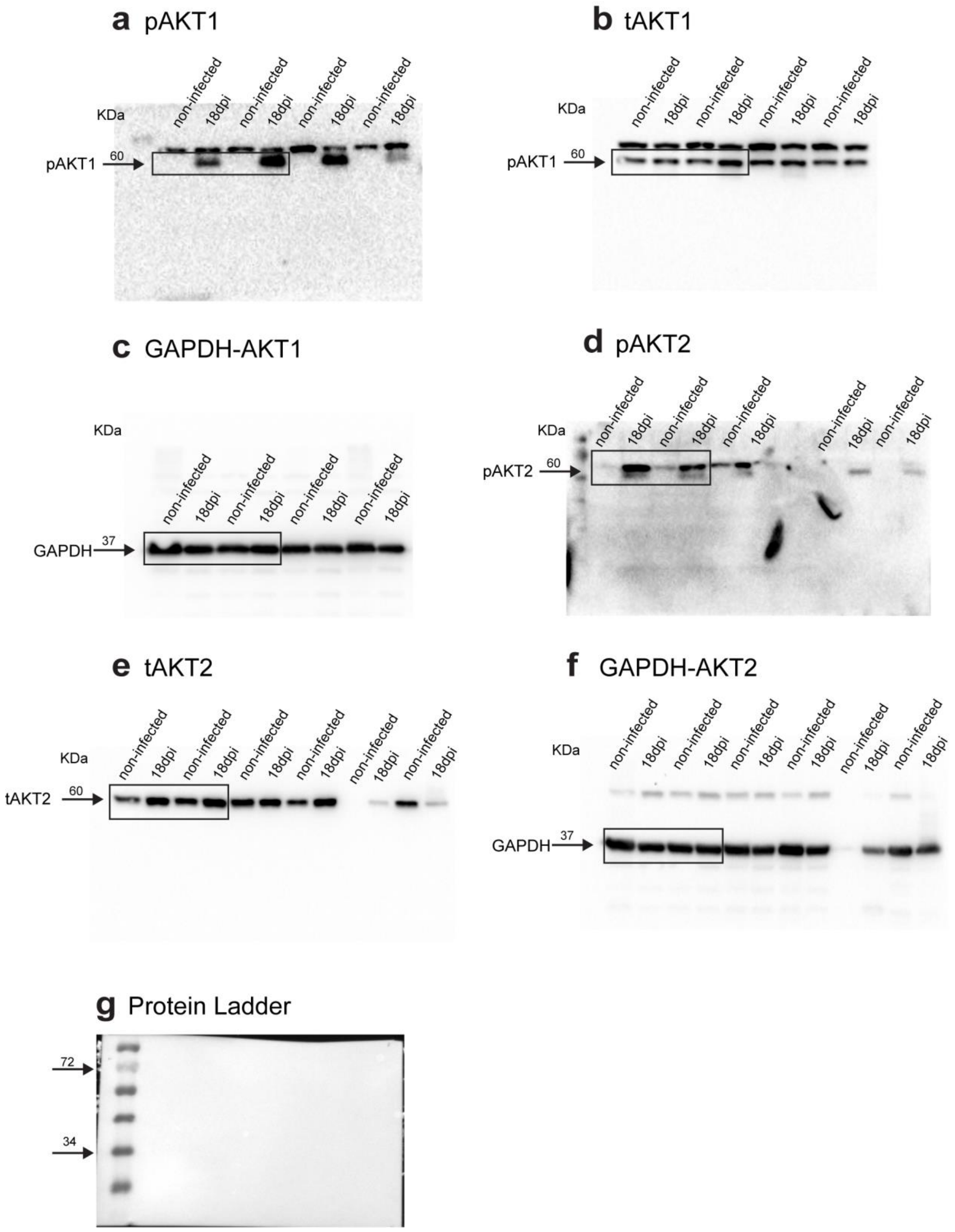

Supplementary Figure 9. Uncropped scans of the western blots presented in main figure 1.

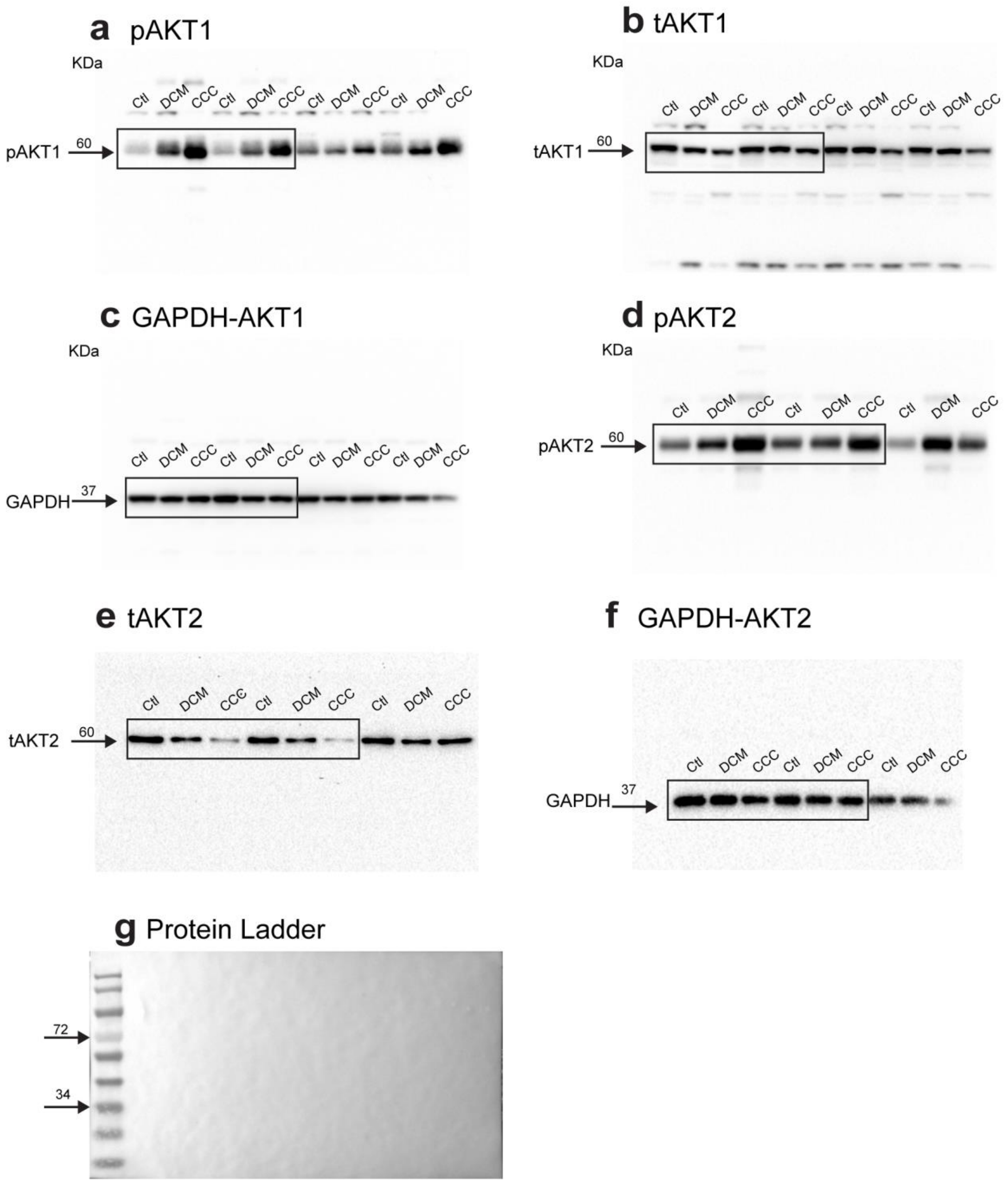

Supplementary Figure 10. Uncropped scans of the western blots presented in main figure 9.

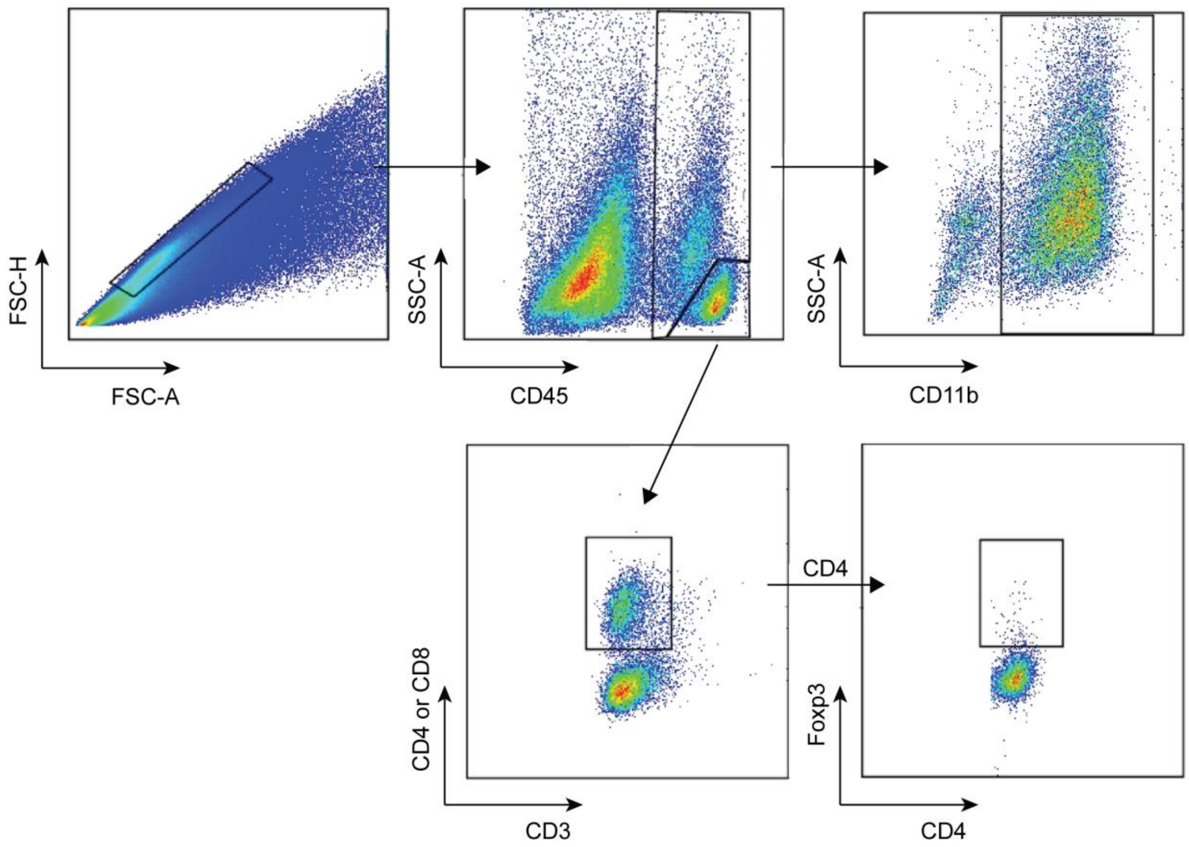

Supplementary Figure 11. Representative gating strategies for flow cytometry analysis. FSC-H/FSC-A preliminary gate was performed for all cytometry analysis to exclude cell debris and cell doublets. Next, $CD45^+$ cells were gated on two populations according their relative complexity (SSC). The percentage of $CD3^+$, $CD4^+$, $CD8^+$ cells was evaluated within the less complex subpopulation and the frequency of $CD11b^+$ cells was evaluated within the most complex cell subpopulation.

Supplementary Table 1 Multiplex analyses of cytokines/chemokines profile in the heart tissue of WT and *Pik3cg*$^{-/-}$ mice after *T. cruzi* infection

	Proinflamatory cytokines (pg g^{-1})							
**Group**	**IL-1β**	**TNF-α**	**IFN-γ**	**IL-6**	**IL-12 (p40)**	**IL12(p70)**	**IL-17**	**IL-1α**
**WT NI**	303.9 ± 75.36	9.478 ± 1.996	543.3 ± 68.42	321.3 ± 32.47	229.8 ± 7.371	52.58 ± 12.55	14.65 ± 2.224	1588 ± 140.6
***Pik3cg*$^{-/-}$ NI**	181.9 ± 29.49	7.377 ± 1.565	259.4 ± 66.68	180.5 ± 8.488	165.3 ± 40.45	36.62 ± 3.041	14.45 ± 0.8474	776.7 ± 134.2
**WT 18dpi**	190.9 ± 46.50	7.156 ± 1.643	1839 ± 199.5	409.0 ± 54.76	419.1 ± 67.39	81.69 ± 17.14	19.32 ± 3.457	2028 ± 547.6
***Pik3cg*$^{-/-}$ 18dpi**	763.3 ± 108.3***$^{\#\#}$	26.38 ± 4.808***$^{\#\#}$	6417 ± 929.7***$^{\#\#\#}$	8192 ± 1908***$^{\#\#\#}$	513.6 ± 105.5	111.3 ± 23.73	20.32 ± 3.969	1848 ± 385.1

	Proinflamatory cytokines (pg g^{-1})				Chemokines (pg g^{-1})			
**Group**	**IL-2**	**IL-7**	**IL-9**	**IL-15**	**MIP-1β**	**MIG**	**Eotaxin**	**IP-10**
**WT NI**	176.4 ± 16.62	37.89 ± 4.388	19870 ± 2902	686.7 ± 59.36	68.69 ± 24.53	7511 ± 1017	425.3 ± 34.17	403.2 ± 27.89
***Pik3cg*$^{-/-}$ NI**	98.55 ± 11.46	27.05 ± 3.003	14520 ± 2443	592.7 ± 92.31	15.26 ± 7.711	6010 ± 263.4	429.2 ± 23.71	346.4 ± 43.40
**WT 18dpi**	88.29 ± 13.05	32.61 ± 3.671	12770 ± 1081	580.1 ± 46.51	22080 ± 1050$^{\#\#\#}$	232800 ± 25090$^{\#\#}$	1834 ± 94.61$^{\#\#\#}$	17840 ± 914.9$^{\#\#\#}$
***Pik3cg*$^{-/-}$ 18dpi**	81.68 ± 9.106	36.91 ± 4.564	18090 ± 5018	661.6 ± 131.3	33730 ± 3535**$^{\#\#\#}$	369300 ± 57980*$^{\#\#\#}$	3255 ± 124.5***$^{\#\#\#}$	35760 ± 2673***$^{\#\#\#}$

	Chemokines (pg g^{-1})						Growth Factors (pg g^{-1})
**Group**	**MIP-1α**	**RANTES**	**LIX**	**MIP-2**	**MCP-1**	**KC**	**G-CSF**
**WT NI**	166.0 ± 102.5	66.02 ± 3.436	1475 ± 264.9	245.3 ± 162.6	591.4 ± 90.30	449.6 ± 34.39	56.29 ± 6.367
***Pik3cg*$^{-/-}$ NI**	461.3 ± 54.81	45.45 ± 4.277	851.8 ± 28.12	429.0 ± 61.00	473.8 ± 47.60	343.1 ± 25.36	41.81 ± 5.944
**WT 18dpi**	3696 ± 255.2$^{\#\#\#}$	17090 ± 7405	1087 ± 179.8	427.5 ± 137.8	10140 ± 991.8	859.9 ± 82.31	74.88 ± 24.75
***Pik3cg*$^{-/-}$ 18dpi**	7789 ± 498.2***$^{\#\#\#}$	90870 ± 41570	3661 ± 178.1***$^{\#\#\#}$	4728 ± 992.6***$^{\#\#\#}$	67310 ± 10770***$^{\#\#\#}$	4180 ± 397.6***$^{\#\#\#}$	1091 ± 150.3***$^{\#\#\#}$

	Growth Factors (pg g^{-1})			Antiinflamatory (pg g^{-1})				
**Group**	**LIF**	**M-CSF**	**VEGF**	**IL-5**	**IL-13**	**IL-3**	**IL-10**	**IL-4**
**WT NI**	28.74 ± 2.688	447.8 ± 75.99	496.3 ± 109.7	66.73 ± 16.32	1023 ± 101.0	20.48 ± 4.059	156.8 ± 13.27	18.51 ± 2.611
***Pik3cg*$^{-/-}$ NI**	27.24 ± 0.9582	151.6 ± 24.52	486.4 ± 24.28	21.18 ± 2.395	822.3 ± 87.52	12.10 ± 1.140	128.8 ± 22.90	12.92 ± 0.9351
**WT 18dpi**	31.50 ± 4.076	277.5 ± 15.43	90.82 ± 29.91$^{\#\#\#}$	36.41 ± 7.510	519.7 ± 182.5	10.72 ± 1.091	83.38 ± 10.37	24.68 ± 1.868
***Pik3cg*$^{-/-}$ 18dpi**	182.2 ± 42.27***$^{\#\#}$	522.3 ± 58.94**$^{\#\#}$	41.32 ± 7.177$^{\#\#\#}$	40.98 ± 8.933	122.5 ± 29.95$^{\#\#}$	14.42 ± 2.429	127.8 ± 47.21	26.93 ± 2.987

*$P<0.05$, **$P<0.01$ and ***$P<0.001$ relative to WT infected group and #$P<0.05$, ##$P<0.01$ and ###$P<0.001$ relative to non-infected group (one-way ANOVA with Tukey's post-hoc test). Mean ± s.e.m.

Supplementary Table 2 Multiplex analyses of cytokines/chemokines profile in the heart tissue of WT and *Pik3cg*$^{KD/KD}$ mice after *T. cruzi* infection

	Proinflamatory cytokines (pg g^{-1})							
**Group**	**IL-6**	**IFN-γ**	**TNF-α**	**IL-1β**	**IL-7**	**IL12(p70)**	**IL-17**	**IL-15**
**WT NI**	321.3 ± 32.47	543.3 ± 68.42	9.478 ± 1.996	303.9 ± 75.36	37.89 ± 4.388	52.58 ± 12.55	14.65 ± 2.224	686.7 ± 59.36
**WT 18dpi**	704.9 ± 193.0	1289 ± 62.53	18.34 ± 8.474	349.9 ± 72.09	51.84 ± 33.02	59.82 ± 30.70	16.55 ± 4.443	647.1 ± 163.0
***Pik3cg*$^{KD/KD}$ 18dpi**	15410 ± 3479**$^{\#\#}$	3647 ± 803.9*$^{\#\#}$	46.21 ± 9.218$^{\#}$	492.3 ± 23.11	18.91 ± 5.447	44.99 ± 8.644	10.48 ± 1.141	488.5 ± 57.68

	Proinflamatory cytokines (pg g^{-1})				Chemokines (pg g^{-1})			
**Group**	**IL-12(p40)**	**IL-2**	**IL-1α**	**IL-9**	**MIG**	**MIP-1β**	**MIP-1α**	**Eotaxin**
**WT NI**	229.8 ± 7.371	176.4 ± 16.62	1588 ± 140.6	19870 ± 2902	7511 ± 1017	68.69 ± 24.53	166.0 ± 102.5	425.3 ± 34.17
**WT 18dpi**	386.5 ± 118.8	57.92 ± 11.93$^{\#\#\#}$	594.3 ± 121.2$^{\#\#\#}$	11510 ± 1345$^{\#}$	212400 ± 53880$^{\#\#}$	38990 ± 21700$^{\#\#\#}$	4066 ± 238.9$^{\#\#\#}$	1592 ± 210.3$^{\#\#}$
***Pik3cg*$^{KD/KD}$ 18dpi**	304.3 ± 56.68	40.47 ± 5.109$^{\#\#\#}$	501.8 ± 34.91$^{\#\#\#}$	12130 ± 2001	189800 ± 26750$^{\#}$	34030 ± 4971$^{\#\#\#}$	7652 ± 243.5***$^{\#\#\#}$	3073 ± 193.2***$^{\#\#\#}$

	Chemokines (pg g^{-1})						Growth factors (pg g^{-1})
**Group**	**IP-10**	**MIP-2**	**MCP-1**	**LIX**	**KC**	**RANTES**	**LIF**
**WT NI**	403.2 ± 27.89	245.3 ± 162.6	591.4 ± 90.30	1475 ± 264.9	449.6 ± 34.39	66.02 ± 3.436	28.74 ± 2.688
**WT 18dpi**	18540 ± 1629$^{\#\#\#}$	694.3 ± 202.6	15950 ± 1389$^{\#\#\#}$	1093 ± 135.4	1249 ± 130.3$^{\#\#}$	19360 ± 4867$^{\#}$	44.34 ± 14.02
***Pik3cg*$^{KD/KD}$ 18dpi**	21850 ± 1596$^{\#\#\#}$	6659 ± 1874**$^{\#\#}$	61920 ± 8352***$^{\#\#\#}$	4672 ± 360.9***$^{\#\#\#}$	5716 ± 764.2***$^{\#\#\#}$	14050 ± 6155	344.7 ± 56.12***$^{\#\#\#}$

	Growth factors (pg g^{-1})			Antiinflammatory (pg g^{-1})				
**Group**	**G-CSF**	**M-CSF**	**VEGF**	**IL-10**	**IL-13**	**IL-5**	**IL-3**	**IL-4**
**WT NI**	56.29 ± 6.367	447.8 ± 75.99	496.3 ± 109.7	156.8 ± 13.27	1023 ± 101.0	66.73 ± 16.32	20.48 ± 4.059	18.51 ± 2.611
**WT 18dpi**	171.0 ± 33.57	365.7 ± 78.93	17.78 ± 3.931$^{\#\#\#}$	129.5 ± 32.46	641.5 ± 185.1	42.72 ± 13.46	11.15 ± 1.423	15.99 ± 3.002
***Pik3cg*$^{KD/KD}$ 18dpi**	8270 ± 2222**$^{\#\#}$	800.5 ± 117.7*	14.35 ± 1.994$^{\#\#\#}$	103.9 ± 15.86	189.9 ± 81.88$^{\#\#}$	39.84 ± 13.88	10.10 ± 1.192	16.64 ± 3.240

*P<0.05, **P<0.01 and ***P<0.001 relative to WT infected group and #P<0.05, ##P<0.01 and ###P<0.001 relative to non-infected group (one-way ANOVA with Tukey's post-hoc test). Mean ± s.e.m.

Supplementary Table 3 List of real-time PCR primers

	Gene	Sequence
**Human**	*PIK3CA*	fwd: 5’- TCAAAGGATTGGGCACTTTT - 3’
		rev: 5’- AATACATCCCACATGCACGA - 3’
	PIK3CB	fwd: 5’- CAGTCAACATCAGCGCAAAG - 3’
		rev: 5’- ACAAGGAAAAACAGGAAATACAGAA - 3’
	PIK3CD	fwd: 5’- AAATTTGAACGGTTCCGGGG - 3’
		rev: 5’- CCTCCTCTGTTTTCCCCAGT - 3’
	PIK3CG	fwd: 5’- CATATTGACTTCGGGCACATTCTTG - 3’
		rev: 5’- GTCTCTGCAAACTTCGATCTGATC - 3’
	TNF	fwd: 5’- AAGCCTCTAGCCCATGTTGT - 3’
		rev: 5’- CAGATAGATGGGCTCATACC - 3’
	IFNG	fwd: 5’- TGCACCTCATTCAGATGTAG - 3’
		rev: 5’- AGCCATCACTTGGATGAGTT - 3’
	CCL5	fwd: 5’- TCATTGCTACTGCCCTCTGC - 3’
		rev: 5’- CGTCGTGGTCAGAATCTGGG - 3’
	GAPDH	fwd: 5’- TGAGTGTGGCAGGGACT - 3’
		rev: 5’- AGGGTGGTGGACCTCAT - 3’
**Mouse**	*Pik3ca*	fwd: 5’- GATAAAGGTTGCCACGCAGT - 3’
		rev: 5’- GCAGTTCAACAGCCACACAC - 3’
	Pik3cb	fwd: 5’- TGAAGCTAGCCCCTGTGACT - 3’
		rev: 5’- GGGCAGGGTTCTATTCATCA - 3’
	Pik3cd	fwd: 5’- CTGGAACAGCCATTCTCCAT - 3’
		rev: 5’- TGACACAGTCTTGCACAGCA - 3’
	Pik3cg	fwd: 5’- ATTGCTCTGGCATTTTCGATA - 3’
		rev: 5’- TACGTTTTGGCAACAATTTCTT - 3’
	T-bet	fwd: 5’- AACCAGTATCCTGTTCCCAGC - 3’
		rev: 5’- TGTCGCCACTGGAAGGATAG - 3’
	Gata-3	fwd: 5’- CTCCTTTTTGCTCTCCTTTTC - 3’
		rev: 5’- AAGAGATGAGGACTGGAGTG - 3’
	Foxp3	fwd: 5’- ACAACCTGAGCCTGCACAAGT - 3’
		rev: 5’- GCCCACCTTTTCTTGGTTTTG - 3’
	Rorγt	fwd: 5’- TGGAAGATGTGGACTTCGTTT - 3’
		rev: 5’- TGGTTCCCCAAGTTCAGGAT - 3’
	T. cruzi	fwd: 5’- AAATAATGTACGGG(T/G)GAGATGCATGA - 3’
		rev: 5’- GGGTTCGATTGGGGTTGGTGT - 3’
	Gapdh	fwd: 5’- GTGGAGTCATACTGGAACATGTAG - 3’
		rev: 5’- AATGGATGAAGGTCGGTGTG - 3’
